# An Overview of Antibiotic Resistance and Abiotic Stresses Affecting Antimicrobial Resistance in Agricultural Soils

**DOI:** 10.3390/ijerph19084666

**Published:** 2022-04-12

**Authors:** Abdullah Kaviani Rad, Siva K. Balasundram, Shohreh Azizi, Yeganeh Afsharyzad, Mehdi Zarei, Hassan Etesami, Redmond R. Shamshiri

**Affiliations:** 1 Department of Soil Science, School of Agriculture, Shiraz University, Shiraz 71946-85111, Iran; arad@adaptiveagrotech.com; 2 Department of Agriculture Technology, Faculty of Agriculture, University Putra Malaysia, Serdang 43400, Selangor, Malaysia; 3 UNESCO-UNISA Africa Chair in Nanosciences and Nanotechnology, College of Graduate Studies, University of South Africa, Pretoria 0003, South Africa; azizis@unisa.ac.za; 4 Nanosciences African Network (NANOAFNET), iThemba LABS-National Research Foundation, Cape Town 7129, South Africa; 5 Department of Microbiology, Faculty of Modern Sciences, The Islamic Azad University of Tehran Medical Sciences, Tehran 19496-35881, Iran; info@ordibeheshtmedlab.com; 6 Department of Agriculture and Natural Resources, Higher Education Center of Eghlid, Eghlid 73819-43885, Iran; 7 Department of Soil Science, University of Tehran, Tehran 14179-35840, Iran; hassanetesami@ut.ac.ir; 8 Leibniz Institute for Agricultural Engineering and Bioeconomy, 14469 Potsdam-Bornim, Germany; rshamshiri@atb-potsdam.de

**Keywords:** antibiotic resistance, antimicrobials, agriculture, livestock, abiotic stress, salinity, heat, soil pollutants, bioremediation

## Abstract

Excessive use of antibiotics in the healthcare sector and livestock farming has amplified antimicrobial resistance (AMR) as a major environmental threat in recent years. Abiotic stresses, including soil salinity and water pollutants, can affect AMR in soils, which in turn reduces the yield and quality of agricultural products. The objective of this study was to investigate the effects of antibiotic resistance and abiotic stresses on antimicrobial resistance in agricultural soils. A systematic review of the peer-reviewed published literature showed that soil contaminants derived from organic and chemical fertilizers, heavy metals, hydrocarbons, and untreated sewage sludge can significantly develop AMR through increasing the abundance of antibiotic resistance genes (ARGs) and antibiotic-resistant bacteria (ARBs) in agricultural soils. Among effective technologies developed to minimize AMR’s negative effects, salinity and heat were found to be more influential in lowering ARGs and subsequently AMR. Several strategies to mitigate AMR in agricultural soils and future directions for research on AMR have been discussed, including integrated control of antibiotic usage and primary sources of ARGs. Knowledge of the factors affecting AMR has the potential to develop effective policies and technologies to minimize its adverse impacts.

## 1. Introduction

As a critical element of ecosystems on the Earth and a tremendous reservoir of microbial diversity, soil has various microbiomes involved in the nutrient cycles, pollution remediation, and production of bioactive compounds, such as antimicrobials that boost humans and animals’ health [1,2,3]. Soil microbial communities have manifold benefits for plants, including nutrient supply, synthesis of phytohormones, antagonistic activity against phytopathogens, and generation of signal molecules involved in microbe–plant interactions [4]. Although microbial communities are indicators of overall soil health, their resistance to adverse conditions for survival and competition is a considerable concern [5] owing to their potential to create ecosystem imbalances and disease emergence [6]. Hence, many studies have been carried out on microbial ecology in the environment [7], and most investigations concentrate on bioactive antimicrobial compounds in fertilizers, soil, and water resources [8]. Antibiotics, as one of the most common antimicrobials and a valuable scientific discovery in the twentieth century, caused a massive shift in pharmaceutical and veterinary sciences [9]. Molecules with antibiotic properties existed prior to humans producing manufactured antibiotics. However, widespread production of these compounds, along with synthetic derivatives of natural antibiotics, began in the twentieth century [10]. Soil actinobacteria and myxobacteria are the most significant fundamental reservoirs of active metabolites. More than 60% of natural antimicrobial compounds belong to actinobacteria and fungi [11].

**Figure 1:**
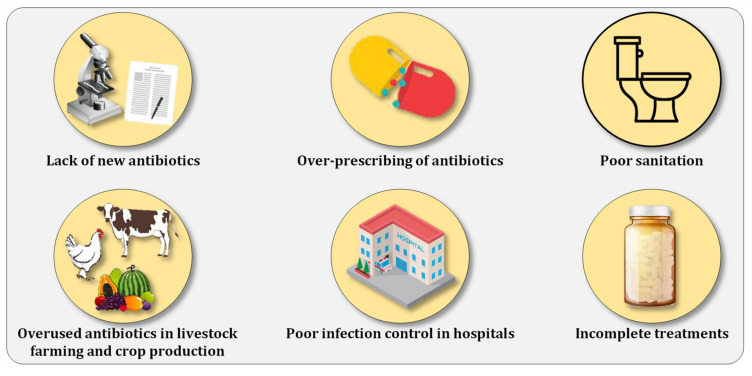
The most consequential causes of AMR, adapted from the World Health Organization (WHO) [25].

Antibiotics are applied to protect human health, inhibit animal disease emergence, and increase the production rate in dairy farms. However, their environmental consequences have recently been reconsidered as a significant concern [12,13,14,15] due to the low absorption of antibiotics in the guts of animals. Approximately 10–90% of these complexes are discharged through urine and feces in a stable form [15,16,17] that creates new sorts of antimicrobial resistance, such as antibiotic-resistant bacteria (ARB) and antibiotic-resistant genes (ARGs) [18,19]. Antimicrobial resistance (AMR) happens when microbes do not perish from intended drugs, thereby making them challenging or inconceivable to control [20]. The indiscriminate use of antibiotics accelerates AMR, leading to higher medical costs and mortality [21]. More than 700,000 people worldwide die each year from AMR, which is predicted to reach 10 million deaths by 2050 and reduce gross domestic product by 8.3% [22]. In this regard, the US Centers for Disease Control and Prevention (CDC) [23] reported that antibiotic-resistant diseases affect approximately 8.2 million Americans every year. Accordingly, one of the top ten worldwide healthcare issues is AMR [24]. Figure 1 summarizes the significant factors involved in the evolution of AMR.

**Figure 2:**
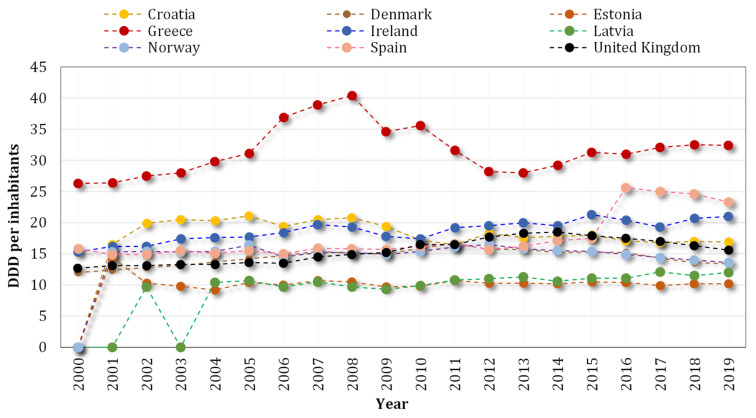
The increasing consumption trend of antibiotics in some European countries from 2000 to 2019. Defined daily dose (DDD) per 1000 inhabitants per day. Data source: [31].

Since ARGs are inserted into the human body via inhalation pathways and consumption of foods manufactured with polluted soils [26], the overuse of antibiotics in the agriculture sector has raised questions concerning various influences of antimicrobial compounds on soil microbiota composition and the risk of AMR expansion [27]. As one of the most important users of antimicrobials, agriculture leads to severe ecological issues in countries such as China, which applies more than 84,000 tons of antibiotics to the dairy farm industry [28,29]. Figure 2 reveals the rising antibiotic usage in several European countries from 2000 to 2019. Although the share of agriculture in AMR is not precisely identified, it is estimated that 50–80% of total antibiotics are used in the agricultural production process [30].

**Figure 3:**
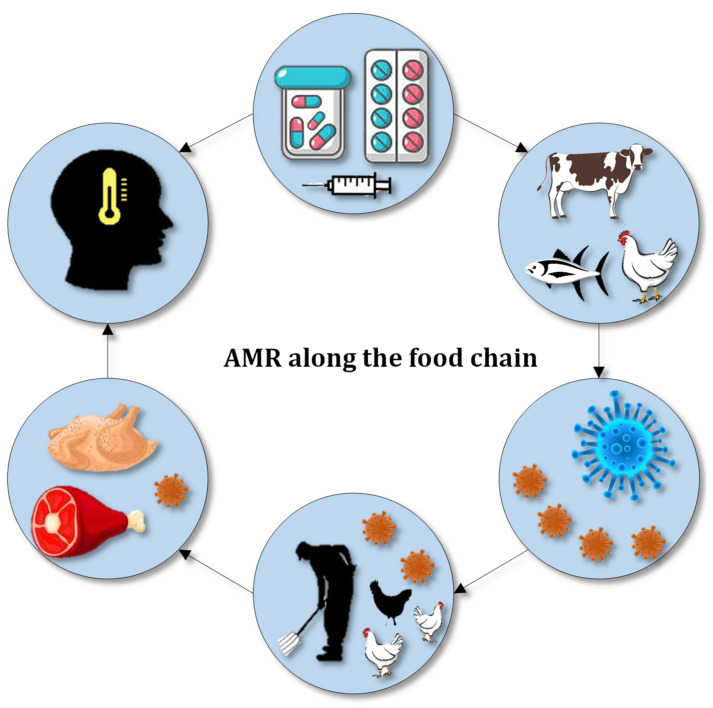
Transmission of AMR agents from soil to the human body through the food chain, adapted from WHO [25].

Agriculture intensifies AMR through three primary mechanisms: (i) creating infection caused by livestock products, (ii) transmission of resistant strains through the food chain, and (iii) carrying ARGs from environmental matrices to the human body [32]. According to a study by Hassan et al. [33], antibiotic residues in the liver and kidneys of poultry, fish, and dairy products such as meat, milk, and eggs can drive AMR in humans via transmission through the food chain and increase damage to bone marrow and reproductive organs [34] (Figure 3). Antibiotics can affect microbial community composition, selection of resistant microorganisms, and bacterial physiology via agricultural activities, including animal manure application, aquaculture, and using untreated wastewater, which drives soil and water resource pollution [35,36]. Antibiotic residues have direct or indirect consequences, including reducing or eliminating microbial communities or expanding ARBs [37]. Degrading the soil microbiome caused by antibiotics can break the food chain due to the elimination of soil nutrients, further affecting microbial processes such as mineralization and decomposition of organic matter [17]. By amplicon sequencing, Lucas et al. [38] recognized that antibiotics changed the stoichiometry of soil nutrients, which lessened bacterial plenty, total available nitrogen, and microbial carbon utilization efficiency. In a study by Toth et al. [39], sulfadimethoxine released from manure blocked iron reduction and had an inhibitory outcome on soil nitrification. Kong et al. [40] found that when 5 mM OTC and 20 mM Cu were combined, the indexes of Shannon diversity and evenness were considerably reduced compared to when the two pollutants were separately applied. Additionally, the use of carbohydrates and carboxylic acids in the soil microbial community significantly decreased. Through employing pyrosequencing methods of 16 S rRNA genes, Uddin et al. [41] evaluated the synergistic effects of several antibiotics on the bacterial communities of a paddy soil sample. They recognized that the abundance of actinobacteria and firmicutes was reduced. It has been reported that reducing biodiversity drives an increase in the transmission of infectious agents [42], resulting in the inhabiting suppression of soil pathogens [43]. However, various influences of antibiotics on the activities of soil microbial communities are still assumed as a significant crux [44].

The ecological effects of antibiotics in soils are not ignorable, even at low concentrations [45]. Accordingly, numerous studies are being conducted on the factors influencing the uptake and stamina of these compounds in soils. Absorption and stabilization of antibiotics in soil particle surfaces depend on physicochemical characteristics of antibiotics, soil pH, colloids, porosity, soil class and texture, organic matter, nutrient availability, and syntrophic or antagonistic organisms [15,46]. Clay particles can absorb antibiotics due to their high surface area [47]. Despite the advantageous properties of clay particles to carry nutrients [48], Sanchez-Cid et al. [49] reported that gentamicin was actively absorbed by clay particles, which inhibited soil bacterial enrichment. The persistence of antibiotics in soils also depends on the uptake of organic particles and the degradation duration [44]. Therefore, the dynamics of ARBs and ARGs in soils, as well as the horizontal gene transfer process, are influenced by soil management systems, oxygen levels, organic carbon, and nutrients [50,51]. The process of transmitting genetic materials across cells is known as “horizontal gene transfer” [52]. New DNA and RNA can substitute existing genes or insert a novel gene into the genome [53], resulting in new functionalities such as environmental adaptability and AMR in the host. Fertilizer administration and irrigation water quality dramatically influence ARG [54] owing to the long half-life and high solubility of antibiotics in waters that enhance their durability [55].

It is known that plants can affect the human gut microbiome, similar to the soil microbiome [56]. Various plant organs and tissues, such as roots and cotyledons, potentially absorb antibiotic residues [15]. Plant roots can be colonized by soil bacteria that are inherent tanks of ARGs [57]. Phytotoxic impacts of antibiotics on several plant species showed that rice was the most susceptible plant to sulfamethoxazole at a dose of 1 mg L^−1^ [58]. In a comparison by Yu et al. [59], the mean concentration of antibiotics in the samples of *Brassica rapa* subsp. seedlings planted in soil polluted with antibiotics was three times higher than of the control group. Tadic et al. [60] identified residues of 16 antibiotics in lettuce, tomato, cauliflower inflorescences, and bean seed over the method detection limit. Kumar et al. [61] determined that the presence of antibiotics in animal manure enhances the concentration of chlortetracycline in the plant tissues of corn, green onions, and cabbage. Hence, consuming vegetables cultivated in soils fertilized with fertilizers containing antibiotics is more hazardous for sensitive bodies. Consequently, the concentration of antibiotics in vegetables is not ignorable [62].

In addition to plants, antibiotics also pose a critical hazard to animal microbiomes. In this regard, Li et al. [63] demonstrated that adding penicillin (100 mg kg^−1^ soil) to the soil decreased the probiotic *Lactobacillus* and sulfate-reducing bacteria and also increased *enterobacteria* and bacteroids, which are resistant to penicillin in the ileum of mice. In research by Dong et al. [64], tetracycline generated significant genetic toxicity in *Eisenia fetida* earthworms after being treated with 3 mg kg^−1^ of tetracycline for 7 days. Yuan et al. [65] observed that amoxicillin treatment dramatically reduced the biodiversity of the *Lactobacillus* species in mice after five weeks. AMR affects the rectal microbiota of aquatic organisms [28]. ARBs can transfer their genes to native microbes in water and conceivably remodel microbiomes [66]. In a study by Xue et al. [67], the infection of water with antibiotics remarkably reduced the bacterial biomass of the *Hemiculter leucisculus* gut. In their study, Qian et al. [68] assessed the impacts of doxycycline, oxytetracycline, and florfenicol residues on adult zebrafish. They found that rectal mucus secretion and microbiota diversity were significantly reduced. The effects of antibiotic residues also cause gut microbiota dysfunction and hepatic metabolic disturbances. Therefore, antibiotics’ potential toxicity, teratogenicity, and genetic toxicity have drawn considerable attention, even for aquatic organisms [9]. Table 1 summarizes several studies on AMR in animals caused by antibiotic overuse.

AMR, as a developing hidden ecological problem in farming soils, has been converted into a significant environmental and health threat worldwide [80,81,82]. Moreover, the lack of awareness of microbial responses lessens soil biodiversity protection in agricultural ecosystems [83]. Increasing evidence points to the crucial role of environmental factors in the transmission process of ARGs [84,85]. Hence, it is essential to cope with AMR in humans and animals via understanding ARG enrichment mechanisms and resistance gene stamina in plants and soil [81]. Due to enhanced selective pressure, the evolution of resistant bacteria has been accelerated in recent years [86], and abiotic stress agents may amplify bacterial resistance to a wide variety of antibiotics [84]. Though abiotic stresses such as salinity, heavy metal accumulation, application of untreated sewage, hydrocarbon pollutants, and irrational use of fertilizers and pesticides are the most critical production challenges in sustainable agriculture, there are few comprehensive studies concerning associations between abiotic stresses and the consequences of antibiotic residues in soils. Presently, abiotic stresses as key limiting factors challenge many farmers’ livelihoods worldwide, with decreasing crop yields ranging from 50% to 70% [87,88]. Hence, this study attempts to review comprehensive interactions between abiotic stresses and antibiotic residues and, consequently, AMR. Keywords such as “antibiotic resistance,” “resistant bacteria,” “antibiotic residues,” “soil pollutants,” “ARGs in wastewater,” “ecosystem and AMR,” and “strategies to combat AMR” were searched in Google Scholar and PubMed databases. Afterward, 296 references were selected and surveyed by the systematic review method. According to a systematic classification, abiotic stresses affecting AMR and strategies to reduce AMR are discussed in Section 2 and Section 3, respectively.

## 2. Abiotic Stresses

### 2.1. Soil Pollutants

#### 2.1.1. Fertilizers

Although fertilizers are unavoidable in order to ensure crop production sustainability and yield enhancement, the excessive application of them potentially drives soil degradation and environmental pollution [89,90]. Cerqueira et al. [91] reported that fertilization imports more ARG into crops than irrigation water. Sun et al. [92], using metagenomics sequencing of soil samples in a vegetable greenhouse, observed that applying both organic and chemical fertilizers, including chicken manure, urea, (NH_4_)_2_HPO_4_, and K_2_(SO_4_), enhances the frequency and diversity of ARGs. Subsequently, Wang et al. [93] recognized that chemical fertilizers such as nitrogen fertilizers [94] had a moderate impact on the diversity of ARGs and a minor effect on the relative enhancement of the abundance of total ARGs. It has even been reported that pesticide applications increase the abundance of ARGs, developing resistant phenotypes to antibiotics [95,96]. Another investigation by Kang et al. [97] showed that applying fresh pig manure as an alternative to agrochemicals increased the chance of spreading tetracycline resistance genes. Both NPK fertilizer and NPK fertilizer + straw return fertilizers decreased soil pH and induced significant variations in bacterial communities, although they moderately affected ARG diversity and abundance. At the same time, the addition of pig manure significantly affected ARG profiles. However, it maintained the diversity of the bacterial community [98].

Despite the fact that organic manures are commonly used to improve soil fertility, using these fertilizers significantly increases ARG abundance when compared to control samples [99]. Zhao et al. [100] found that more than 99% of the antibiotics in organic manures are released into the soil–plant system. The presence of seven trace elements, including Cu, Zn, As, Cr, Hg, Pb, and Cd, as well as four antibiotic combinations, sulfonamides, tetracycline, fluoroquinolones, and chloramphenicol, was identified in the organic manures of Zhejiang Province, China, by Qian et al. [101]. The mean concentrations of metals were 160, 465, 7.9, 21.2, 0.3, 8.1, and 0.6 mg kg^−1^, respectively. An analysis of pig manure in vitro conditions in Austria indicated that the fertilizers added chlortetracycline, enrofloxacin, and ciprofloxacin to the soil [102]. Ruuskanen et al. [103] determined that the relative abundance of ARGs increased almost four-fold after applying cattle and pig manure to some farms in southern Finland. In a study by Liu et al. [104], the application of chemical and organic fertilizers enabled a decrease in the abundance of *Gaiella* from 12.9% in non-fertilized soil to 4.1–7.4% in fertilized soil (*p* < 0.05). A significant rise in copies of the sulfonamide resistance gene (*sul2*) was recognized after treating soil samples with organic manure [105]. Wei et al. [106] indicated that oxytetracycline, chlortetracycline, enrofloxacin, and ciprofloxacin have drastic ecological hazards in soils. Genes *sul2*, *sul1*, *oqxA*, *qnrs*, *tetB*, *tetA*, *ermaA*, and *floR* were detected in strains resistant to tetracycline, quinolones, sulfonamides, and macrolides. Han et al. [107] similarly observed that in the soils treated with organic manure carrying chlortetracycline and ciprofloxacin, the abundance of tetracycline-resistant genes *tet X*, *tet X2*, *tet A* (G), *tet W*, *tet A*, *tet A* (33), and *tet A* (P) increased. Liu et al. [104] demonstrated that fertilizers could considerably alter bacterial communities and affect soil resistome composition. Ma et al. [108] mentioned that the abundance of planctomycetes was dramatically decreased from 33.05% to 3.28% after 14 days of exposure to tetracycline. Liu et al. [109] similarly reported that the functional diversity of a paddy loam soil microbial community was reduced after seven days of sulfamethoxazole exposure. It was demonstrated that long-term grazing is frequently correlated with inserting manure into rangelands and changes in the diversity and composition of bacterial communities [110]. Zhou et al. [111] determined that applying commercial manure-based fertilizers impressively enhanced the relative abundance of ARGs in soils.

In an analysis by Liu et al. [112], the diversity of ARGs in soils fertilized with pig, poultry, and cattle manure increased during three consecutive years. ARGs have been proven to be closely connected with the number of mobile gene elements (MGEs) and bacteria. Prokaryotic cells can spread characteristics such as AMR by transmitting their DNA to other cells using MGEs [113]. According to Solliec et al. [114], pig manure inserts an extensive array of veterinary antibiotics into agricultural soils. These researchers identified the presence of tetracyclines, beta-lactams, sulfonamides, and lincosamides in pig manure samples. The soil microbiome generated doxycycline resistance genes along with adding pig manure containing doxycycline [115]. Chen et al. [116] reported that the soils treated with pig manure had more ARG diversity. Since livestock manures are known as primary reservoirs of antibiotics and ARBs [117] and the application of chemical fertilizers is increasing due to agricultural development, it is expected to understand the long shelf life of ARGs in soil ecosystems following fertilizer utilization [118]. Although attempts have been made to determine manure application rates, the inaccessibility of accurate data from farms has caused investigations to be impossible [119].

#### 2.1.2. Heavy Metals

Although AMR is often attributed to selective stress due to organic manure overuse, evidence reveals that chemicals also stimulate AMR [120]. In a study by Kang and So [121], AMR patterns in ureolytic bacteria revealed that resistance to heavy metals in these bacteria is significantly correlated with their resistance to antibiotics. Most previous investigations have focused on fertilizer-derived ARGs; however, it is known that heavy metals can also induce the simultaneous selection of resistance-determining genes and ARGs in bacteria [122]. Nutrients, heavy metals, and bacterial communities are directly and indirectly involved in ARG discharge [123]. It was further pointed out that heavy metals enhance ARGs’ proliferation through co-selection, and Ag^+3^ ions considerably increased ARGs and altered their attributes in soil [124]. Evidence collected by Yang et al. [125] explained that antibiotics and the simultaneous selection of heavy metals were the chief determinants in releasing ARGs in six urban lakes in China. A significant correlation (*p* < 0.05) was recognized between some ARGs and heavy metals [126]. Lu et al. [127] observed a positive association between ARGs/antibiotics and heavy metals in Lake Chanshou (China), which indicates the potential effect of heavy metals on ARGs. Moreover, Zhou et al. [128] recognized that ARGs and metal resistance genes (MRGs) were dramatically associated with heavy metals in dung (*p* < 0.01). Heavy metals induce metal resistance as well as selection processes of ARGs. The presence of heavy metals was associated with a 2.67-fold and a 3.86-fold increase in *tetG* and *sul1*, which accelerated ARG diffusion. Additionally, a remarkable relationship was detected between ARGs and Cu, corresponding with its high toxicity [129]. By studying three commercial poultry farms, Mazhar et al. [130] ascertained that the metals Cd, As, Zn, Cu, and Pb had the highest positive connections with ARGs in comparison to antibiotics. Therefore, metals had a more significant impact on ARG profiles than detected antibiotics.

Presumably, heavy metals participate in antibiotic-resistant strains’ co-selection in ecosystems. According to the research carried out by Nguyen et al. [131], Zn and Cd were the most frequent heavy metals correlated with AMR. *Pseudomonas aeruginosa* and *Escherichia coli* were the most prevalent bacteria with synchronous resistance to heavy metals and antibiotic collections. Seiler and Berendonk [132] demonstrated that the heavy metals Hg, Cd, Cu, and Zn were presumably capable of creating the co-selection of AMR. The most prevalent AMR pattern was witnessed in agricultural soils contaminated with amoxicillin, ampicillin, streptomycin, vancomycin, tetracycline, and doxycycline. Furthermore, a high level of synchronous resistance to Hg and antibiotics was recorded among Gram-negative isolates against Zn, Ni, and Hg against beta-lactam antibiotics among Gram-positive isolates [133]. Yamamura et al. [134] pointed to As-resistant bacteria as a shielded strain versus multiple antibiotics. In a study by Chen et al. [135], the presence of As, Cu, and Zn strengthened the resistance of a bacterium with the LSJC7 gene sequence to tetracycline. The negligible content of heavy metals in polluted ecosystems and treated organisms may be adequate to stimulate AMR since Zn, Ag, and Cu enhance the gene mutation rate and enrich antibiotic-resistant mutant bacteria even at sub-lethal levels [136]. Moreover, Zhang et al. [120] revealed that low concentrations of Cr, Ag, Cu, and Zn extend the horizontal transfer of ARGs, and also have adverse effects on beneficial soil microbiomes. A gradient gel electrophoresis of soil samples isolated from seven farms in China by Zhang et al. [137] demonstrated that the most abundant bacterial species were in soils with the least heavy metal pollution. Xing and Jin [138] concluded that the toxicity of Zn^+2^ and Cu^+2^ plus oxytetracycline and sulfamethazine synergistically inhibits the activity of nitrifying bacteria. Table 2 summarizes several investigations into simultaneous resistance to antibiotics and heavy metals.

#### 2.1.3. Hydrocarbons

**Figure 4:**
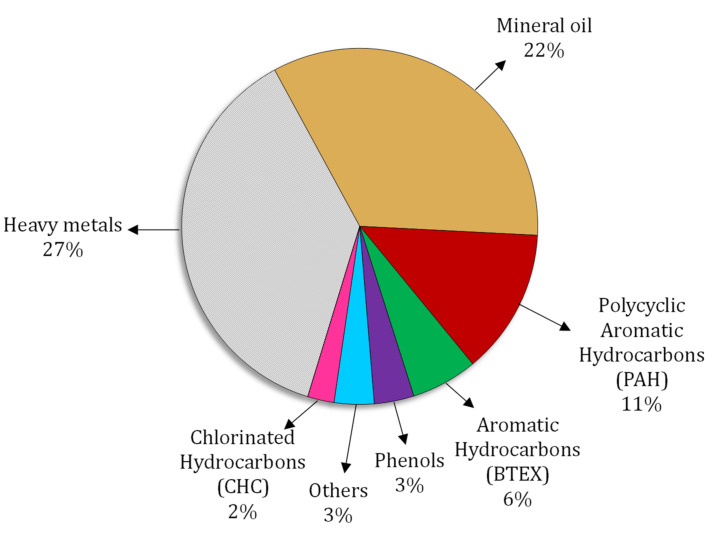
The main pollutants in soils, adapted from the European Environment Agency [165].

Industrialization and the consequential uncontrolled discharge of pollutants directly influence soil health, ecosystems, and eventually human health [148]. It is known that petroleum hydrocarbons induce the emergence of ARBs and ARGs in soils, encouraging researchers to promote precision monitoring instruments and evaluate ARG transmission and fate [149]. Polycyclic aromatic hydrocarbons (PAHs) cause significant alterations in soil microbiomes and enhance the abundance of actinobacteria, which carry multiple ARGs. PAH-polluted soils are potentially a selective environment for antibiotic-resistant bacteria owing to high ARG expression levels [122]. The effects of naphthalene and phenanthrene on extending AMR in a coastal microbial community were investigated by Wang et al. [150], who demonstrated that the presence of 100 mg L^−1^ of naphthalene or 10 mg L^−1^ of phenanthrene significantly increased the frequency of the class I integrase (*intl1*) gene, sulfanilamide resistance gene (*sul1*), and aminoglycoside resistance gene (*aadA2*) in microbial communities. A study on metagenomics profiles of soil samples from three petrochemical plant zones confirmed that ARGs were 15-fold more abundant in PAH-polluted soils. *Proteobacteria* selected by PAHs resulted in the simultaneous enrichment of ARGs. It was also shown that PAHs could operate as selective stresses, enriching ARGs in ecosystems influenced by human activities [151]. Amala et al. [152] remarked that hydrocarbon soot caused by incomplete combustion of fossil fuels directed resistance in *Staphylococcus aureus* and *E. coli* isolates. Antibiotic-resistant bacteria may thrive in hydrocarbon-contaminated soils due to selective pressures [153]. The ARG abundance of fluoroquinolones in PAH-contaminated soils was ten times higher than control samples in an examination by Das et al. [154]. Analyzing soil samples isolated from an industrial site in the Alps showed that approximately half of the 47 strains isolated from the soil were resistant to penicillin [155]. “Bisphenol A” is a widely known synthetic compound used in the production of polycarbonate plastics and epoxy resins [156,157]. Evidence reveals that bisphenol A, which accumulates in animal tissue, negatively impacts the endocrine system [158,159]. In this regard, Eladak et al. [160] reported that bisphenol S and bisphenol F decrease testosterone secretion in humans. Russell [161] found a relationship between bisphenol and triclosan contamination with AMR in *E. coli*. Hartmann et al. [162] also demonstrated a significant association between triclosan and the *erm(X)* gene in the dust microbiome. Therefore, it is necessary to implement risk assessment programs for emerging soil pollutants such as PAHs, bisphenol, and triclosan in order to dominate ARGs in ecosystems [163,164]. Figure 4 reveals the contribution of various hydrocarbon contaminants to soil pollution.

In addition to PAHs, the extensive production and use of plastics as petroleum derivatives in recent decades has increased the amount of plastic waste entering the environment [166]. Microplastics (MPs), as emerging contaminants, are potential carriers of pathogenic bacteria and enrichment factors of ARBs [167]. Moreover, the accumulation of ecological contaminants and bacterial communities on MPs in wastewater treatment plants and soils drives the transmission of ARGs [168]. The primary reservoirs of MPs are the composting process, mulching, application of polymer-containing pesticides, and irrigation with wastewater [169]. Growing evidence reveals that MPs could absorb antibiotics, posing multiple hazards to organisms’ health [170]. In research by Yan et al. [171], ARGs belonging to tetracycline, beta-lactam, and sulfonamide were identified on plastic surfaces. Accordingly, MPs transfer the ARGs deep into the soil. Huang et al. [172] demonstrated that MPs significantly increase the abundance of ARGs in sediments. According to a comparison by Peng et al. [173], microplastic polymers had a positive relationship with the relative abundance of ARGs. Wang et al. [174] also investigated the impacts of mixing tetracycline, Cu, and MPs in soil and concluded that the abundance of ARGs in the soil can increase by 219–348%. Furthermore, MPs may enhance the stimulatory effects of Cu plus tetracycline on AMR. In an examination by Shi et al. [175], MPs had more ARGs than landfill leachate, and 11 pathogens were recognized. Additionally, polyethylene scraps in wastewater operate as potential resistant microbiota vectors [176].

As potential habitats for pathogens, MPs intervene in various metabolic pathways that directly drive soil ecological processes [177]. Sathicq et al. [178] similarly pointed to MPs as a unique ecological locality that assists the survival of pathogens and ARBs, further enhancing horizontal gene transfer. Horizontal gene transfer between exiting microbes on MP surfaces is more active than in free-living microbes [179]. MPs can increase the uptake of pollutants by plants. Consequently, more experiments are required to examine the long-term hazards of soil MPs [180]. Given that soil contamination with MPs has been less considered than pollution of water sources, an increasing investigation trend into the outcomes of MPs on soil ecosystems in the future is predicted [181]. Zhang et al. [182] also recommended that future studies on the consequences of soil MPs should address issues of the global distribution of soil MPs and their disadvantageous influences on soil organisms.

#### 2.1.4. Sewage Sludge

Reusing wastewater is a practical solution for watering crops in order to cope with water deficiencies and nutrients, especially in arid and semi-arid regions. As a hotspot emitting ARGs, sewage sludge is one of the most critical potential hazards [183,184,185]. Sewage sludge, as a source of high organic carbon, lipids, and nutrients [186], carries many beneficial microorganisms [187]. However, it contains antibiotics, disinfectant chemicals, and metals that induce simultaneous exposure of prokaryotic communities to antibiotics and heavy metals in agriculture [188] and ultimately drive selective pressure toward extending AMR [189]. Using a high-throughput sequencing metagenomics approach, Yang et al. [190] identified 14 varieties of ARGs in sewage sludge. Some AMR-related genes such as *catB3*, *catB3*, *catA1*, *sul1*, and *qnrD* were detected in soil samples treated with sewage sludge in Ontario [191]. In the research by Lyu et al. [192], the presence of tetracyclines and quinolones in soil samples was attributed to applying fertilizer and domestic wastewater. Parallel Shotgun Sequencing of a bacterial community of irrigated soil and lettuce root samples containing trimethoprim, ofloxacin, and sulfamethoxazole identified a total of 56 ARGs, which were resistant to 14 classes of antibiotics [193].

**Figure 5:**
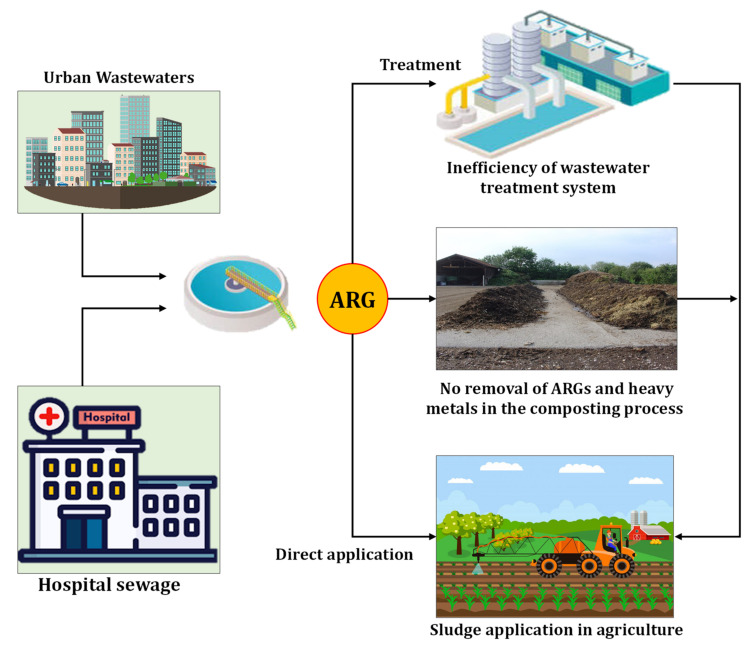
The routes of ARGs from hospitals and urban wastewater to farmlands.

Antibiotic residues and other medical compounds enter the environment directly due to inefficient treatment processing of hospital sewage or urban wastewater [194,195]. Hubeny et al. [196] recognized wastewater treatment systems as foci of AMR. The results obtained by Rahube et al. [197] revealed that crop production in lands fertilized with human waste without appropriate pretreatment techniques drives an extra load of ARGs into crops. Zarfel et al. [198] hypothesized that population transferring between human infectious *E. coli* and beta-lactamase-producing bacteria transpires in sewage treatment systems. Sahlström et al. [199] probed sludge samples of a treatment plant in Sweden and demonstrated that applying sewage sludge for fertilizing purposes prompts vancomycin-resistant *enterococci* in agricultural ecosystems. Markowicz et al. [200] concluded that utilizing sewage sludge might induce public health concerns, even at low doses. The routes of entry of ARGs from urban and hospital sewage to farmlands are shown in Figure 5.

Considering the interactions between antibiotics, heavy metals, and ARGs in sewage sludge, the use of sewage sludge in agricultural soils is controversial [201], though it meets the demands of farm soils for organic matter and nutrients [202]. The long-term consequences of sewage sludge containing pharmaceutical compounds on soil attributes are not entirely realized. However, European Commission strategies are shifting towards enhancing the reuse of sewage sludge in farming [203]. Although the European Commission has established several constraints on the presence of heavy metal contaminants in sewage sludge, governments are obtaining new reports on contaminants in sewage sludge worldwide [204]. Therefore, more legal ordinances to manage environmental pollutants in countries that utilize sewage sludge in farming are required [205]. Improving disinfection methods and combating resistant bacteria caused by hospital sewage are critical for limiting AMR [66]. Further studies should concentrate on presumptive human health threats in sewage sludge such as heavy metals, PAHs, nanoparticles, phenols, pesticides, as well as antibiotics [206].

### 2.2. Salinity

As a critical abiotic stress, salinity significantly restricts crop production in agriculture [207]. The comparative abundance of total ARGs was significantly correlated with soil salinity characteristics such as electrical conductivity (EC), sodium, and chloride [84]. In a study by Yan et al. [208], enhancing salinity by 1% neutralized the threat of MGEs by eliminating *Pseudomonas* and *Methylophilus*, which are resistant to sulfamethoxazole and naproxen; thereby, the total diversity and abundance of ARGs in biofilms were considerably decreased. Tan et al. [209] remarked that along with increasing soil depth and lowering EC, the quantity of ARGs and MGEs grows, indicating a reduction of ARGs in saline soils correlated with the relative decline of plasmid-containing strains carrying ARG.

High salt concentrations in mangrove soil samples separated from the rhizosphere of a forest zone in Colombia altered metabolic pathways. Furthermore, 16 of the 33 genes involved in intrinsic AMR were significantly impressed by salinity [210]. Liu et al. [211] demonstrated that adding NaCl to a wastewater treatment reactor reduced the relative abundance of genes *tetG*, *sul2*, and *amrB* by roughly 50%, which was logically due to inhibiting the growth of some bacteria carrying ARGs. Moreover, enhancing salinity led to a 24–33% decline in the bioaccumulation of sulfamethoxazole in zebrafish (*Danio rerio*) [212]. Additionally, in an investigation by Yang et al. [213], along with the increasing salinity of seawater, the toxicity of sulfonamide antibiotics for bioluminescent bacteria and *Vibrio fischeri* was lowered. Salinity has been shown to be the most significant element in modulating ARG distribution patterns in coastal soils [86].

## 3. Combating Antibiotic Resistance Prevalence

Presumably, the most helpful and cost-effective solution to diminish the development of AMR is to optimize the use of antibiotics [214]. Flawless monitoring of antibiotic application in hospitals is a promising approach to declining human-resistant pathogens [215]. Antibiotic management policies must be executed effectively, despite the fact that antibiotics are essential in safeguarding animal health and financial benefits [216]. The antibiotic control policy between the 1990s and 2000s transformed the pig meat industry in Denmark. Notwithstanding the adverse economic consequence of decreasing antibiotics in the pork industry, a considerable decline in the abundance of vancomycin-resistant *enterococci* was detected in pigs’ guts [217]. Furthermore, the Dutch mandatory policy to reduce antibiotic usage in the livestock sector in 2008 reduced beta-lactams, aminoglycosides, fluoroquinolones, and tetracycline consumption by roughly 50% by 2013 [218]. In the United States, the Food and Drug Administration (FDA) strategy decreased sales of primary veterinary antibiotics by approximately 43% between 2015 and 2017, whereas meat production remained unaffected [219]. Although governments must execute the policies of the Food and Agriculture Organization (FAO) and WHO to prevent the occurrence of AMR in agricultural soils [220], reducing antibiotic consumption may not be attainable in existing circumstances due to economic losses for growers [221]. Implementing precision agriculture approaches as an alternative to intensive farming is presumably an ideal method to decrease AMR. Since evidence demonstrates that intensive agriculture has a substantial share in antibiotic usage [222], for instance, approximately 3558–4279 tons of antimicrobials were applied in the livestock sector in Africa from 2015 to 2019; consequently, a high level of AMR was detected in farming soils [223]. Hence, precision agriculture enables administrators to maintain the health of the environment and agricultural sustainability by controlling fertilizer consumption as AMR causes and sustaining crop yield, leading to higher financial profitability and ensuring food security [224,225,226].

Controlling the sources of antibiotic remnants through treating urban wastewater and organic manure is another practical method to ameliorate the unfavorable impacts of antibiotics on agricultural ecosystems [227]. Since antibiotics are resistant to degradation due to their hydrophobic and lipophilic properties [228], traditional wastewater treatment processes cannot completely eliminate ARGs [26]. Therefore, it is necessary to employ additional techniques to improve the expulsion of ARGs. Recently, to enhance wastewater treatment efficiency, clay minerals having high adsorption traits, easy availability, and low cost have been regarded as alternatives to activated carbon in removing metal ions [229,230,231]. Mustapha et al. [232] pointed to kaolin as an effective adsorbent for removing chloride, sulfate, Cr, Cd, and Zn. In addition, Yang et al. [233] reported that kaolin increased the removal efficiency of naproxen and diclofenac from water. Clays can split heavy metals from water, although their adsorption efficiency depends on the metal concentration, pH, ion type, surface area, and adsorbent dose [234]. Hence, more research into the commercial use of clay is required. Salts can be applied for the biological treatment of wastewater comprising antibiotics [208] by inhibiting the growth of bacteria containing ARGs. Hence, using salts such as sodium chloride can be a viable method to reduce ARGs in wastewater systems [211]. Heating beef for 30 min reduces antibiotic residues by 10.8% [235], and Zhang et al. [236] also documented that oxidation of heat-activated persulfate residues of erythromycin could restrict the spread of MGEs; hence, heating is a potentially efficient method to diminish ARGs in sewage sludge. Thermophilic anaerobic digestion of urban sewage sludge in an analysis by Xu et al. [237] led to a 29.59% decline in *Proteobacteria* and 17.65% in *Chloroflexi,* as well as more effective elimination of tetracycline-, macrolide-, and fluoroquinolone-resistant genes. Thermal hydrolysis treatment of wastewater sludge also lessened ARGs and MGEs, tetracyclines, macrolides, and lincosamides by 94% [238]. In a study by Liao et al. [239], hyperthermophilic composting removed ARGs and MGEs by 89% and 49% more than traditional composting methods, respectively.

The composting operation helps with a decrease in pathogenic bacteria activities and ARGs, enriching the soil’s beneficial microbiome and nutrients. The diversity of potential pathogenic bacteria declined from 37.18% to 3.43%, and probiotic species were enhanced from 5.77% to 7.12% during the composting process [240]. Gou et al. [241] indicated that levels of AMR in compost-treated soils were reduced, and compost remarkably lowered the relative diversity and abundance of ARGs and MGEs in cattle dung. Analysis of livestock manure containing 16 sorts of antibiotics showed that the composting process significantly reduced extractable antibiotics [242]. An experiment conducted by Keenum et al. [243] revealed that the composting operation reduces the risk of AMR spreading; however, it cannot prevent whole ARGs from penetrating the soil. Sardar et al. [244] considered conventional composting an inefficient technique to control AMR owing to a considerable increase in the abundance of *sul1* and *sul2* genes after 30 days of the initial composting phase. Additionally, Gao et al. [245] demonstrated that composting poses a prominent risk to human health by emitting bioaerosol pollutants containing ARGs into the atmosphere. At the same time, composting was an appropriate mechanism for removing polycyclic aromatic hydrocarbons such as chrysene and indenopyrene from soil [246]. Moreover, bioremediation, which employs microbial-base catabolic processes to environmental pollutants’ degradation [247,248], can be adopted as an efficient method to ameliorate the health of hydrocarbon-contaminated soils [249]. In an examination by Ghazali et al. [250], a consortium of *Bacillus* and *Pseudomonas* species effectively decomposed medium-chain alkanes in diesel-contaminated soil. Gargouri et al. [251] revealed that the bacterial consortium effectively eliminated long-chain alkanes in contaminated soil after 30 days. It also reduced total petroleum hydrocarbon (TPH) from 63.4 to 2.5 mg g^−1^. In an experiment by Guarino et al. [252], soil bioremediation with a bacterial consortium decreased TPHs by 86%.

Numerous bacteria can remove soil pollutants and antibiotic residues from ecosystems by bioremediation. Sulfate-Reducing Bacteria (SRB) are a diverse group of prokaryotes that can grow in various environmental conditions [253,254] and are a cost-effective, eco-friendly, and helpful method for the bioremediation processes of pollutants from wastewater and contaminated soils [255]. In a study by Jong and Parry [256], SRBs increased the removal of sulfate and heavy metals such as Cu, Zn, and Ni by elevating the pH of water from 4.5 to 7. Zhao et al. [257] found that SRB strains such as *Desulfobacteraceae* and *Desulfovibrionaceae* had a considerable impact on Cd immobilization in sediments. SRBs can also be used to purify soils contaminated with benzene, toluene, and xylene [258]. It has been reported that SRBs can effectively remove the antibiotic ciprofloxacin from wastewater [259,260]. In a study by Zhang et al. [261], it was demonstrated that approximately 35% of the total removal of ciprofloxacin from wastewater was related to SRB. Jia et al. [262] also reported that *Desulfobacter* decomposed nearly 28% of the 5000 μg L^−1^ ciprofloxacin concentration. In a study by Zhang et al. [263], *Arthrobacter nicotianae* OTC-16 was detected as an oxytetracycline biodegradation instrument. Maki et al. [264] demonstrated that microbes might be involved in the degradation of ampicillin, doxycycline, and oxytetracycline residues in marine fish farm sediments. In research by Hirth et al. [265], *Microbacterium* sp. increased the elimination of sulfamethazine residues in soil by 44% after 46 days. In addition, Mojiri et al. [266] reported that marine diatoms were able to decompose 39.8% of sulfamethoxazole and 42.5% of ofloxacin in an aqueous environment. While bioremediation of antibiotic residues is a promising and cost-effective method, more knowledge is required regarding the mechanisms of microbial degradation of antibiotics and the potential undesirable hazards of microbes in ecosystems [267]. Furthermore, the biodegradation of antibiotics in soils is highly conditional on microbiomes, pH, temperature, and different interactions among antibiotics [46].

The microbial electrolysis cell system (MECs), as an emerging contamination control technology, can diminish the release of ARGs [268]. Through analyzing the effects of microbial electrolysis cells on the decomposition of erythromycin in wastewater, Hua et al. [269] demonstrated that electrolysis is an effective technique to enhance the decomposition efficiency of antibiotics. Microbial electrolysis by Zhang and Li [270] in a sewage sludge treatment plant with a voltage range of 0 to 1.5 V removed most of the targeted ARGs. The results obtained by Zhang and Li [271] indicated that the removal efficiency of antibiotics at a voltage of 0.6 to 1 V was higher than at other voltages. In addition to microbial metabolism and electrochemical redox reactions, bioelectrochemical systems are promising alternatives for decomposing antibiotic residues [272]. Moreover, controlling the discharge of antibiotics into the environment should be characterized according to their adverse impacts [273].

The use of natural antibiotics is one of the most effective ways to reduce AMR. Investigations to discover natural alternatives to synthetic antibiotics have advanced in recent years, and antioxidants such as polyphenols, vitamins, and carotenoids have garnered considerable attention due to their antibacterial and anti-inflammatory activities [274,275]. Herbal antibiotics, which are derived from plants such as garlic and aloe vera, have fewer side effects than conventional antibiotics [276]. Some plant-derived compounds, such as quinine and artemisinin, have actively combated infectious diseases [277]. Fit et al. [278] examined the impact of plant extracts on pathogenic strains of *S. aureus* isolated from animal waste. They demonstrated that savory and fir had antibacterial activity. Awan et al. [279] reported that chloroform extract of cumin and turmeric had significant antibacterial activity against *Serratia marcescens* and *P. aeruginosa*. Saquib et al. [280] documented that combining the antibiotic metronidazole with the ethanolic extract of the Miswak plant had a synergistic influence against *Aggregatibacter actinomycetemcomitans*. Nweze and Eze [281] also reported that the ethanolic extract of lamiaceae leaves mixed with ampicillin had a synergistic effect against *E. coli* and *Candida albicans*. At the same time, it is recommended that more clinical trials be conducted on the effectiveness of plant-derived antibiotics to combat AMR [282,283]. In addition to herbal antibiotics, animals also contain peptides with antimicrobial functions [284]. Antimicrobial peptides (AMPs) are cationic and amphipathic peptides that are important in the natural defense of organisms and can be isolated from all kingdoms [285,286]. AMPs, such as defensins and cathelicidins, can eliminate bacteria by creating pores in the phospholipid membrane and disrupting its integrity, thereby diminishing AMR development [287,288,289]. Hence, the identification and optimization of AMPs have attracted ample attention, and more than 2493 AMPs have been documented in 2014 [290,291]. Despite the fact that AMPs appear to be a promising alternative for combating AMR due to their ability to damage pathogenic bacteria’s membranes, it is critical to investigate their structural changes and potential side effects on animal and human physiology [292,293]. AMR is gradually progressing into an unmanageable condition [294], and there are no geographical boundaries to stop its expansion as an international challenge [295]. Accordingly, global collaboration is needed to battle the imminent AMR crisis [296].

## 4. Conclusions

Antibiotics are used to safeguard human health and prevent the spread of animal diseases. However, uncontrolled antibiotic use coupled with the increasing trend of industrialization and agribusiness development, and subsequently, the entry of various contaminants into the environment, leads to AMR, an unavoidable phenomenon that significantly imperils the health of organisms. Presently, infections caused by ARBs lead to the deaths of thousands of people each year. Furthermore, the entry of antibiotic residues into the soil reduces the abundance and diversity of beneficial soil microbial communities that are effective in ecosystem balance. Promoting livestock and aquaculture production, in addition to sewage sludge application for fertilizing agricultural soils, are critical elements in developing AMR since they transmit ARGs from primary sources such as hospitals and urban sewage to farmlands. It is known that abiotic stresses such as salinity and soil and water pollutants, which have a negative effect on agriculture production, can affect AMR in soils. According to the literature review, hydrocarbons, heavy metals, and untreated sewage sludge can significantly increase AMR. Conversely, some evidence has demonstrated that salinity and heat stress effectively decrease the abundance and diversity of ARGs. Therefore, more statistical investigations are needed into the role of abiotic stresses in developing or declining AMR. Given the fact that slight attention has been paid to the consequences of cold, waterlogging, drought, and radioactive stresses on AMR, more examinations should be conducted regarding the role of various environmental stresses on AMR in agricultural soils.

Considering the growing world population and, accordingly, the increasing demand for livestock production, antibiotics, and countering the spread of AMR, it is crucial to shift policies for controlling antibiotic consumption toward precision agriculture approaches that prevent the overuse of agricultural inputs such as fertilizers and pesticides, as well as technologies such as bioremediation and microbial electrolysis that remove antibiotic residues prior to joining water resources and soils. Heavy metals and hydrocarbons can be extracted from soil and wastewater using clay and bacteria such as kaolin and SRB. Further studies should be carried out on natural plant-derived antibiotics, or AMPs, that slow the development of AMR. Given that antibiotic management policies have been successfully implemented in some countries, it is necessary to adopt (i) an integrated global strategy in order to control the usage of antibiotics in hospitals and the livestock sector and (ii) advantageous methods such as optimizing wastewater treatment systems, composting, and bioremediation of soil contaminants to fight AMR as an expanding crisis.

## Figures and Tables

**Table 1:** Some studies on AMR in animals treated with antibiotics.

Antibiotic	Animal	Resistant Bacteria	Result	Ref
Cefalotin, streptomycin, and sulfamethoxazole	Cattle and sheep	*Escherichia coli*	Most isolates were moderately resistant to antibiotics.	[69]
Streptomycin, gentamycin, tetracycline, and trimethoprim	Sheep, goat, camel	*Acinetobacter baumannii*	Antibiotic resistance was observed in more than half of the strains isolated from sheep samples.	[70]
Norfloxacin and Doxycycline	Fowl	*Escherichia coli*	An increasing resistance rate of *E. coli* toward norfloxacin in chickens was detected.	[71]
Ampicillin, tetracycline, and sulfamethoxazole	Broiler	*Escherichia coli*	Isolated strains were resistant to antibiotics.	[72]
Lincomycin, erythromycin, ciprofloxacin, and tetracycline	Wild bird	*Enterococcus faecium*, *Enterococcus hirae*, *Enterococcus durans*, *Enterococcus casseliflavus*	The highest resistance was recorded for lincomycin.	[73]
Ampicillin, tetracycline, and nitrofurantoin	Fish	Gram-negative bacteria	Maximum resistance was recognized for ampicillin and tetracycline.	[74]
Ampicillin, tetracycline, and chloramphenicol	Hen eggshells	*Salmonella enterica*	Most isolates were resistant to ampicillin.	[75]
Tetracycline	Cattle	Gut microbiomes	Resistance to tetracycline was highly prevalent in cattle.	[76]
Tetracycline and clindamycin	Swine	*Staphylococcus* *aureus*	High antibiotic resistance was observed for tetracycline or clindamycin.	[77]
Ciprofloxacin, nitrofurantoin, trimethoprim, and cefalotin	Sheep	*Escherichia coli*	The highest AMR was recorded toward ciprofloxacin (69.4%).	[78]
Ampicillin and tetracycline	Catfish (*Clarias gariepinus*)	*Klebsiella pneumoniae*	All coliform bacteria were resistant to antibiotics.	[79]

**Table 2:** Some studies regarding multiple resistances of bacterial strains to antibiotics and heavy metals.

Strain	Heavy Metal	Antibiotic	Location	Result	Ref
*Pseudomonas putida*, *Staphylococcus epidermidis*, *Serratia ficaria*, and *Bacillus anthracis*	Cu, Cd, Cr, Ag, and Hg	Amoxicillin, gentamycin, vancomycin, tetracycline, and ciprofloxacin	Marchica, Morocco	Simultaneous resistance to heavy metals and antibiotics	[139]
*Aeromonas hydrophila*	Cu, Co, Zn, and Hg	Sulfamide, oxytetracycline, and trimethoprim	Tunisia	Relationship between antibiotic resistance and resistance to heavy metals	[140]
*Pseudomonas aeruginosa*, *Actinomyces turicensis*, and *Micrococcus* sp.	Hg, Cd, Co, Ni, and Cr	Chloramphenicol, streptomycin, erythromycin, and metronidazole	Nigeria	22 out of 270 strains of isolated bacteria had simultaneous resistance to antibiotics and heavy metals	[141]
*Staphylococcus aureus*, *Alcaligenes* sp., *Bacillus* sp. and *Klebsiella* sp.	Pb, Cr, Zn, and Cd	Ampicillin, cefalotin, gentamycin, and doxycyclin	Algeria	Eighty-five percent of heavy metal isolates were similarly resistant to several antibiotics	[142]
*Pseudomonas fluorescens*	Pb, Cu, Cr, Zn, and Hg	Amoxicillin, cefradine, norfloxacin, and tetracycline	Guangzhou, China	Correlation between the antibiotic type and the concentration of heavy metals	[143]
138 halophilic bacterial isolates	Cd, Zn, Pb, Cu, and Co	Cefalexin, vancomycin, cefalotin, and ampicillin	Red Sea, Egypt	Simultaneous resistance to heavy metals and antibiotics	[144]
*Escherichia coli*	Ni, Cr, Cu, Pb, and Cd	-	Yamuna, India	A higher level of metal resistance was recognized by increasing the average concentration of metals	[145]
*Enterococci faecalis*	Zn, Ni, Cu, and Co	Penicillin, ampicillin, ciprofloxacin, and sulfamethoxazole	Iran	Simultaneous resistance to antibiotics and metals in the most strains	[146]
*Staphylococcus aureus*	Pb, Cu, Zn, Cr, Cd, and Ni	Tetracycline, ceftazidime, ciprofloxacin, and vancomycin	Nigeria	Multiple resistance to antibiotics and heavy metals in the strains	[147]

## Data Availability

Data are contained within the article.

## References

[1] Zhu Y.G., Zhao Y., Zhu D., Gillings M., Penuelas J., Ok Y.S., Capon A., Banwart S. (2019). Soil biota, antimicrobial resistance and planetary health. Environ. Int..

[2] Armalyte J., Skerniskyte J., Bakiene E., Krasauskas R., Siugzdiniene R., Kareiviene V., Kerziene S., Klimiene I., Suziedeliene E., Ruzauskas M. (2019). Microbial Diversity and Antimicrobial Resistance Profile in Microbiota from Soils of Conventional and Organic Farming Systems. Front. Microbiol..

[3] Bahram M., Hildebrand F., Forslund S.K., Anderson J.L., Soudzilovskaia N.A., Bodegom P.M., Bengtsson-Palme J., Anslan S., Coelho L.P., Harend H. (2018). Structure and function of the global topsoil microbiome. Nature.

[4] Nelkner J., Henke C., Lin T.W., Patzold W., Hassa J., Jaenicke S., Grosch R., Puhler A., Sczyrba A., Schluter A. (2019). Effect of Long-Term Farming Practices on Agricultural Soil Microbiome Members Represented by Metagenomically Assembled Genomes (MAGs) and Their Predicted Plant-Beneficial Genes. Genes.

[5] Nguyen B.T., Chen Q.L., He J.Z., Hu H.W. (2020). Microbial regulation of natural antibiotic resistance: Understanding the protist-bacteria interactions for evolution of soil resistome. Sci. Total Environ..

[6] Foo J.L., Ling H., Lee Y.S., Chang M.W. (2017). Microbiome engineering: Current applications and its future. Biotechnol. J..

[7] Ochoa-Hueso R. (2017). Global Change and the Soil Microbiome: A Human-Health Perspective. Front. Ecol. Evol..

[8] Kemper N. (2008). Veterinary antibiotics in the aquatic and terrestrial environment. Ecol. Indic..

[9] Bilal M., Mehmood S., Rasheed T., Iqbal H.M.N. (2020). Antibiotics traces in the aquatic environment: Persistence and adverse environmental impact. Curr. Opin. Environ. Sci. Health.

[10] Larsson D.G. (2014). Antibiotics in the environment. Ups. J. Med. Sci..

[11] Durand G.A., Raoult D., Dubourg G. (2019). Antibiotic discovery: History, methods and perspectives. Int. J. Antimicrob. Agents.

[12] Baguer A.J., Jensen J., Krogh P.H. (2000). Effects of the antibiotics oxytetracycline and tylosin on soil fauna. Chemosphere.

[13] Isaacson R.E., Torrence M.E. (2002). The Role of Antibiotics in Agriculture: This Report Is Based on a Colloquium Sponsored by the American Academy of Microbiology Held November 2–4, 2001, in Santa Fe, New Mexico.

[14] Callaway T.R., Lillehoj H., Chuanchuen R., Gay C.G. (2021). Alternatives to Antibiotics: A Symposium on the Challenges and Solutions for Animal Health and Production. Antibiotics.

[15] Akram R., Amin A., Hashmi M.Z., Wahid A., Mubeen M., Hammad H.M., Fahad S., Nasim W. (2017). Fate of Antibiotics in Soil. Antibiotics and Antibiotics Resistance Genes in Soils.

[16] Sarmah A.K., Meyer M.T., Boxall A.B. (2006). A global perspective on the use, sales, exposure pathways, occurrence, fate and effects of veterinary antibiotics (VAs) in the environment. Chemosphere.

[17] Kumar K., Gupta S., Chander Y., Singh A.K. (2005). Antibiotic Use in Agriculture and Its Impact on the Terrestrial Environment. Adv. Agron..

[18] Shen Y., Stedtfeld R.D., Guo X., Bhalsod G.D., Jeon S., Tiedje J.M., Li H., Zhang W. (2019). Pharmaceutical exposure changed antibiotic resistance genes and bacterial communities in soil-surface- and overhead-irrigated greenhouse lettuce. Environ. Int..

[19] Zalewska M., Blazejewska A., Czapko A., Popowska M. (2021). Antibiotics and Antibiotic Resistance Genes in Animal Manure—Consequences of Its Application in Agriculture. Front. Microbiol..

[20] CDC About Antibiotic Resistance, 2021, Last Updated: 13 December 2021. https://www.cdc.gov/drugresistance/about.html.

[21] WHO (2020). Antibiotic Resistance. https://www.who.int/news-room/fact-sheets/detail/antibiotic-resistance.

[22] World Bank (2021). Antimicrobial Resistance (AMR). https://www.worldbank.org/en/topic/health/brief/antimicrobial-resistance-amr.

[23] CDC (2021). 2019 AR Threats Report. https://www.cdc.gov/drugresistance/biggest-threats.html.

[24] WHO (2021). Antimicrobial Resistance. https://www.who.int/news-room/fact-sheets/detail/antimicrobial-resistance.

[25] WHO (2015). Infographics: Antibiotic Resistance. https://apps.who.int/mediacentre/events/2015/world-antibiotic-awareness-week/infographics/en/index.html.

[26] He Y., Yuan Q., Mathieu J., Stadler L., Senehi N., Sun R., Alvarez P.J.J. (2020). Antibiotic resistance genes from livestock waste: Occurrence, dissemination, and treatment. NPJ Clean Water.

[27] Laconi A., Mughini-Gras L., Tolosi R., Grilli G., Trocino A., Carraro L., Di Cesare F., Cagnardi P., Piccirillo A. (2021). Microbial community composition and antimicrobial resistance in agricultural soils fertilized with livestock manure from conventional farming in Northern Italy. Sci. Total Environ..

[28] Yukgehnaish K., Kumar P., Sivachandran P., Marimuthu K., Arshad A., Paray B.A., Arockiaraj J. (2020). Gut microbiota metagenomics in aquaculture: Factors influencing gut microbiome and its physiological role in fish. Rev. Aquac..

[29] Xie W.Y., Shen Q., Zhao F.J. (2017). Antibiotics and antibiotic resistance from animal manures to soil: A review. Eur. J. Soil Sci..

[30] Ter Kuile B.H., Kraupner N., Brul S. (2016). The risk of low concentrations of antibiotics in agriculture for resistance in human health care. FEMS Microbiol. Lett..

[31] ECDC (2020). Country Overview of Antimicrobial Consumption. https://www.ecdc.europa.eu/en/antimicrobial-consumption/database/country-overview.

[32] Chang Q., Wang W., Regev-Yochay G., Lipsitch M., Hanage W.P. (2015). Antibiotics in agriculture and the risk to human health: How worried should we be?. Evol. Appl..

[33] Hassan M.M., El Zowalaty M.E., Lundkvist A., Jarhult J.D., Khan Nayem M.R., Tanzin A.Z., Badsha M.R., Khan S.A., Ashour H.M. (2021). Residual antimicrobial agents in food originating from animals. Trends Food Sci. Technol..

[34] Treiber F.M., Beranek-Knauer H. (2021). Antimicrobial Residues in Food from Animal Origin-A Review of the Literature Focusing on Products Collected in Stores and Markets Worldwide. Antibiotics.

[35] Martinez J.L. (2017). Effect of antibiotics on bacterial populations: A multi-hierachical selection process. F1000Research.

[36] Quaik S., Embrandiri A., Ravindran B., Hossain K., Al-Dhabi N.A., Arasu M.V., Ignacimuthu S., Ismail N. (2020). Veterinary antibiotics in animal manure and manure laden soil: Scenario and challenges in Asian countries. J. King Saud Univ. Sci..

[37] Grenni P., Ancona V., Barra Caracciolo A. (2018). Ecological effects of antibiotics on natural ecosystems: A review. Microchem. J..

[38] Lucas J.M., Sone B.M., Whitmore D., Strickland M.S. (2021). Antibiotics and temperature interact to disrupt soil communities and nutrient cycling. Soil Biol. Biochem..

[39] Toth J.D., Feng Y., Dou Z. (2011). Veterinary antibiotics at environmentally relevant concentrations inhibit soil iron reduction and nitrification. Soil Biol. Biochem..

[40] Kong W.D., Zhu Y.G., Fu B.J., Marschner P., He J.Z. (2006). The veterinary antibiotic oxytetracycline and Cu influence functional diversity of the soil microbial community. Environ. Pollut..

[41] Uddin M., Chen J., Qiao X., Tian R., Arafat Y., Yang X. (2019). Bacterial community variations in paddy soils induced by application of veterinary antibiotics in plant-soil systems. Ecotoxicol. Environ. Saf..

[42] Keesing F., Belden L.K., Daszak P., Dobson A., Harvell C.D., Holt R.D., Hudson P., Jolles A., Jones K.E., Mitchell C.E. (2010). Impacts of biodiversity on the emergence and transmission of infectious diseases. Nature.

[43] Wall D.H., Nielsen U.N., Six J. (2015). Soil biodiversity and human health. Nature.

[44] Cycon M., Mrozik A., Piotrowska-Seget Z. (2019). Antibiotics in the Soil Environment-Degradation and Their Impact on Microbial Activity and Diversity. Front. Microbiol..

[45] Gao F., Cui S., Li P., Wang X., Li M., Song J., Li J., Song Y. (2018). Ecological toxicological effect of antibiotics in soil. IOP Conf. Ser. Earth Environ. Sci..

[46] Zhi D., Yang D., Zheng Y., Yang Y., He Y., Luo L., Zhou Y. (2019). Current progress in the adsorption, transport and biodegradation of antibiotics in soil. J. Environ. Manage..

[47] Chander Y., Kumar K., Goyal S.M., Gupta S.C. (2005). Antibacterial activity of soil-bound antibiotics. J. Environ. Qual..

[48] Merino D., Tomadoni B., Salcedo M.F., Mansilla A.Y., Casalongué C.A., Alvarez V.A. (2020). Nanoclay as Carriers of Bioactive Molecules Applied to Agriculture. Handb. Nanomater. Nanocomposites Energy Environ. Appl..

[49] Sanchez-Cid C., Guironnet A., Wiest L., Vulliet E., Vogel T.M. (2021). Gentamicin Adsorption onto Soil Particles Prevents Overall Short-Term Effects on the Soil Microbiome and Resistome. Antibiotics.

[50] Wang F., Tiedje J.M. (2020). Antibiotic Resistance in Soil. Antibiotic Resistance in the Environment.

[51] Wang F., Xu M., Stedtfeld R.D., Sheng H., Fan J., Liu M., Chai B., Soares de Carvalho T., Li H., Li Z. (2018). Long-Term Effect of Different Fertilization and Cropping Systems on the Soil Antibiotic Resistome. Environ. Sci. Technol..

[52] Soucy S.M., Huang J., Gogarten J.P. (2015). Horizontal gene transfer: Building the web of life. Nat. Rev. Genet..

[53] Keeling P.J., Palmer J.D. (2008). Horizontal gene transfer in eukaryotic evolution. Nat. Rev. Genet..

[54] Cerqueira F., Matamoros V., Bayona J., Pina B. (2019). Antibiotic resistance genes distribution in microbiomes from the soil-plant-fruit continuum in commercial Lycopersicon esculentum fields under different agricultural practices. Sci. Total Environ..

[55] Tasho R.P., Cho J.Y. (2016). Veterinary antibiotics in animal waste, its distribution in soil and uptake by plants: A review. Sci. Total Environ..

[56] Hirt H. (2020). Healthy soils for healthy plants for healthy humans: How beneficial microbes in the soil, food and gut are interconnected and how agriculture can contribute to human health. EMBO Rep..

[57] Wang C., Hu R., Strong P.J., Zhuang W., Huang W., Luo Z., Yan Q., He Z., Shu L. (2021). Prevalence of antibiotic resistance genes and bacterial pathogens along the soil-mangrove root continuum. J. Hazard. Mater..

[58] Liu F., Ying G.G., Tao R., Zhao J.L., Yang J.F., Zhao L.F. (2009). Effects of six selected antibiotics on plant growth and soil microbial and enzymatic activities. Environ. Pollut..

[59] Yu X., Liu X., Liu H., Chen J., Sun Y. (2019). The accumulation and distribution of five antibiotics from soil in 12 cultivars of pak choi. Environ. Pollut..

[60] Tadic D., Bleda Hernandez M.J., Cerqueira F., Matamoros V., Pina B., Bayona J.M. (2021). Occurrence and human health risk assessment of antibiotics and their metabolites in vegetables grown in field-scale agricultural systems. J. Hazard. Mater..

[61] Kumar K., Gupta S.C., Baidoo S.K., Chander Y., Rosen C.J. (2005). Antibiotic uptake by plants from soil fertilized with animal manure. J. Environ. Qual..

[62] Gudda F.O., Waigi M.G., Odinga E.S., Yang B., Carter L., Gao Y. (2020). Antibiotic-contaminated wastewater irrigated vegetables pose resistance selection risks to the gut microbiome. Environ. Pollut..

[63] Li M.Y., Chen X.Q., Wang J.Y., Wang H.T., Xue X.M., Ding J., Juhasz A.L., Zhu Y.G., Li H.B., Ma L.Q. (2021). Antibiotic exposure decreases soil arsenic oral bioavailability in mice by disrupting ileal microbiota and metabolic profile. Environ. Int..

[64] Dong L., Gao J., Xie X., Zhou Q. (2012). DNA damage and biochemical toxicity of antibiotics in soil on the earthworm *Eisenia fetida*. Chemosphere.

[65] Yuan J., Wei H., Zeng B., Tang H., Li W., Zhang Z. (2010). Impact of neonatal antibiotic treatment on the biodiversity of the murine intestinal Lactobacillus community. Curr. Microbiol..

[66] Baquero F., Martinez J.L., Canton R. (2008). Antibiotics and antibiotic resistance in water environments. Curr. Opin. Biotechnol..

[67] Xue X., Jia J., Yue X., Guan Y., Zhu L., Wang Z. (2021). River contamination shapes the microbiome and antibiotic resistance in sharpbelly (*Hemiculter leucisculus*). Environ. Pollut..

[68] Qian M., Wang J., Ji X., Yang H., Tang B., Zhang H., Yang G., Bao Z., Jin Y. (2021). Sub-chronic exposure to antibiotics doxycycline, oxytetracycline or florfenicol impacts gut barrier and induces gut microbiota dysbiosis in adult zebrafish (*Daino rerio*). Ecotoxicol. Environ. Saf..

[69] Goncuoglu M., Bilir Ormanci F.S., Ayaz N.D., Erol I. (2010). Antibiotic resistance of *Escherichia coli* O157:H7 isolated from cattle and sheep. Ann. Microbiol..

[70] Askari N., Momtaz H., Tajbakhsh E. (2019). Acinetobacter baumannii in sheep, goat, and camel raw meat: Virulence and antibiotic resistance pattern. AIMS Microbiol..

[71] Guo K., Zhao Y., Cui L., Cao Z., Zhang F., Wang X., Peng Z., Feng J., Hu T., Dai M. (2021). Longitudinal Surveillance and Risk Assessment of Resistance in *Escherichia coli* to Enrofloxacin from A Large-Scale Chicken Farm in Hebei, China. Antibiotics.

[72] Han T., Zhang Q., Liu N., Wang J., Li Y., Huang X., Liu J., Wang J., Qu Z., Qi K. (2020). Changes in antibiotic resistance of *Escherichia coli* during the broiler feeding cycle. Poult. Sci..

[73] Stepien-Pysniak D., Hauschild T., Dec M., Marek A., Urban-Chmiel R. (2019). Clonal Structure and Antibiotic Resistance of Enterococcus spp. from Wild Birds in Poland. Microb. Drug Resist..

[74] Bhat M.D., Vijay Kumar D., Kumar B., Shetty V., Chakraborty A., Karunasagar I. (2019). Occurrence of antibiotic resistance among Gram negative bacteria isolated from effluents of fish processing plants in and around Mangalore. Int. J. Environ. Health Res..

[75] Fernandez Marquez M.L., Burgos M.J., Pulido R.P., Galvez A., Lopez R.L. (2017). Biocide Tolerance and Antibiotic Resistance in Salmonella Isolates from Hen Eggshells. Foodborne Pathog. Dis..

[76] Lim S.K., Kim D., Moon D.C., Cho Y., Rho M. (2020). Antibiotic resistomes discovered in the gut microbiomes of Korean swine and cattle. Gigascience.

[77] Pexara A., Solomakos N., Govaris A. (2020). Occurrence, antibiotic resistance and enteroxigenicity of Staphylococcus spp. in tonsils of slaughtered pigs in Greece. Lett. Appl. Microbiol..

[78] Eltai N., Al Thani A.A., Al-Hadidi S.H., Abdfarag E.A., Al-Romaihi H., Mahmoud M.H., Alawad O.K., Yassine H.M. (2020). Antibiotic resistance profile of commensal *Escherichia coli* isolated from healthy sheep in Qatar. J. Infect. Dev. Ctries.

[79] Adinortey C.A., Aheto D.W., Boateng A.A., Agbeko R. (2020). Multiple Antibiotic Resistance-Coliform Bacteria in Some Selected Fish Farms of the Central Region of Ghana. Scientifica.

[80] Wang J., Wang L., Zhu L., Wang J., Xing B. (2020). Antibiotic resistance in agricultural soils: Source, fate, mechanism and attenuation strategy. Crit. Rev. Environ. Sci. Technol..

[81] Tyrrell C., Burgess C.M., Brennan F.P., Walsh F. (2019). Antibiotic resistance in grass and soil. Biochem. Soc. Trans..

[82] Smalla K., Cook K., Djordjevic S.P., Klumper U., Gillings M. (2018). Environmental dimensions of antibiotic resistance: Assessment of basic science gaps. FEMS Microbiol. Ecol..

[83] Jiao S., Chen W., Wei G. (2019). Resilience and Assemblage of Soil Microbiome in Response to Chemical Contamination Combined with Plant Growth. Appl. Environ. Microbiol..

[84] Zhang Y.J., Hu H.W., Yan H., Wang J.T., Lam S.K., Chen Q.L., Chen D., He J.Z. (2019). Salinity as a predominant factor modulating the distribution patterns of antibiotic resistance genes in ocean and river beach soils. Sci. Total Environ..

[85] Hill K.E., Top E.M. (1998). Gene transfer in soil systems using microcosms. FEMS Microbiol. Ecol..

[86] Beceiro A., Tomas M., Bou G. (2013). Antimicrobial resistance and virulence: A successful or deleterious association in the bacterial world?. Clin. Microbiol. Rev..

[87] Calanca P.P. (2017). Effects of Abiotic Stress in Crop Production. Quantification of Climate Variability, Adaptation and Mitigation for Agricultural Sustainability.

[88] Francini A., Sebastiani L. (2019). Abiotic Stress Effects on Performance of Horticultural Crops. Horticulturae.

[89] Nyamangara J., Kodzwa J., Masvaya E.N., Soropa G. (2020). The role of synthetic fertilizers in enhancing ecosystem services in crop production systems in developing countries. The Role of Ecosystem Services in Sustainable Food Systems.

[90] Bhattacharya A. (2019). Global Climate Change and Its Impact on Agriculture. Changing Climate and Resource Use Efficiency in Plants.

[91] Cerqueira F., Matamoros V., Bayona J.M., Berendonk T.U., Elsinga G., Hornstra L.M., Pina B. (2019). Antibiotic resistance gene distribution in agricultural fields and crops. A soil-to-food analysis. Environ. Res..

[92] Sun Y., Qiu T., Gao M., Shi M., Zhang H., Wang X. (2019). Inorganic and organic fertilizers application enhanced antibiotic resistome in greenhouse soils growing vegetables. Ecotoxicol. Environ. Saf..

[93] Wang F., Han W., Chen S., Dong W., Qiao M., Hu C., Liu B. (2020). Fifteen-Year Application of Manure and Chemical Fertilizers Differently Impacts Soil ARGs and Microbial Community Structure. Front. Microbiol..

[94] Wang F., Chen S., Wang Y., Zhang Y., Hu C., Liu B. (2018). Long-Term Nitrogen Fertilization Elevates the Activity and Abundance of Nitrifying and Denitrifying Microbial Communities in an Upland Soil: Implications for Nitrogen Loss from Intensive Agricultural Systems. Front. Microbiol..

[95] Curutiu C., Lazar V., Chifiriuc M.C. (2017). Pesticides and antimicrobial resistance: From environmental compartments to animal and human infections. New Pesticides and Soil Sensors.

[96] Malagon-Rojas J.N., Parra Barrera E.L., Lagos L. (2020). From environment to clinic: The role of pesticides in antimicrobial resistance. Rev. Panam. Salud. Publica.

[97] Kang Y., Hao Y., Shen M., Zhao Q., Li Q., Hu J. (2016). Impacts of supplementing chemical fertilizers with organic fertilizers manufactured using pig manure as a substrate on the spread of tetracycline resistance genes in soil. Ecotoxicol. Environ. Saf..

[98] Xie W.-Y., Yuan S.-T., Xu M.-G., Yang X.-P., Shen Q.-R., Zhang W.-W., Su J.-Q., Zhao F.-J. (2018). Long-term effects of manure and chemical fertilizers on soil antibiotic resistome. Soil Biol. Biochem..

[99] McKinney C.W., Dungan R.S., Moore A., Leytem A.B. (2018). Occurrence and abundance of antibiotic resistance genes in agricultural soil receiving dairy manure. FEMS Microbiol. Ecol..

[100] Zhao F., Chen L., Yen H., Sun L., Li S., Li M., Feng Q., Yang L. (2020). Multimedia mass balance approach to characterizing the transport potential of antibiotics in soil-plant systems following manure application. J. Hazard. Mater..

[101] Qian M., Wu H., Wang J., Zhang H., Zhang Z., Zhang Y., Lin H., Ma J. (2016). Occurrence of trace elements and antibiotics in manure-based fertilizers from the Zhejiang Province of China. Sci. Total Environ..

[102] Martinez-Carballo E., Gonzalez-Barreiro C., Scharf S., Gans O. (2007). Environmental monitoring study of selected veterinary antibiotics in animal manure and soils in Austria. Environ. Pollut..

[103] Ruuskanen M., Muurinen J., Meierjohan A., Parnanen K., Tamminen M., Lyra C., Kronberg L., Virta M. (2016). Fertilizing with Animal Manure Disseminates Antibiotic Resistance Genes to the Farm Environment. J. Environ. Qual..

[104] Liu P., Jia S., He X., Zhang X., Ye L. (2017). Different impacts of manure and chemical fertilizers on bacterial community structure and antibiotic resistance genes in arable soils. Chemosphere.

[105] Heuer H., Kopmann C., Binh C.T., Top E.M., Smalla K. (2009). Spreading antibiotic resistance through spread manure: Characteristics of a novel plasmid type with low %G + C content. Environ. Microbiol..

[106] Wei R., He T., Zhang S., Zhu L., Shang B., Li Z., Wang R. (2019). Occurrence of seventeen veterinary antibiotics and resistant bacterias in manure-fertilized vegetable farm soil in four provinces of China. Chemosphere.

[107] Han L., Cai L., Zhang H., Long Z., Yu Y., Fang H. (2019). Development of antibiotic resistance genes in soils with ten successive treatments of chlortetracycline and ciprofloxacin. Environ. Pollut..

[108] Ma J., Zhu D., Chen Q.L., Ding J., Zhu Y.G., Sheng G.D., Qiu Y.P. (2019). Exposure to tetracycline perturbs the microbiome of soil oligochaete Enchytraeus crypticus. Sci. Total Environ..

[109] Liu F., Wu J., Ying G.G., Luo Z., Feng H. (2012). Changes in functional diversity of soil microbial community with addition of antibiotics sulfamethoxazole and chlortetracycline. Appl. Microbiol. Biotechnol..

[110] Du S., Shen J.-P., Sun Y.-F., Bai Y.-F., Pan H., Li Y., Wang Z.-W., Han G.-D., Zhang L.-M., He J.-Z. (2020). Grazing does not increase soil antibiotic resistome in two types of grasslands in Inner Mongolia, China. Appl. Soil Ecol..

[111] Zhou X., Qiao M., Wang F.H., Zhu Y.G. (2017). Use of commercial organic fertilizer increases the abundance of antibiotic resistance genes and antibiotics in soil. Environ. Sci. Pollut. Res. Int..

[112] Liu W., Ling N., Guo J., Ruan Y., Wang M., Shen Q., Guo S. (2021). Dynamics of the antibiotic resistome in agricultural soils amended with different sources of animal manures over three consecutive years. J. Hazard. Mater..

[113] Frost L.S., Leplae R., Summers A.O., Toussaint A. (2005). Mobile genetic elements: The agents of open source evolution. Nat. Rev. Microbiol..

[114] Solliec M., Roy-Lachapelle A., Sauve S. (2015). Development of a suspect and non-target screening approach to detect veterinary antibiotic residues in a complex biological matrix using liquid chromatography/high-resolution mass spectrometry. Rapid Commun. Mass Spectrom..

[115] Yan Q., Li X., Ma B., Zou Y., Wang Y., Liao X., Liang J., Mi J., Wu Y. (2018). Different Concentrations of Doxycycline in Swine Manure Affect the Microbiome and Degradation of Doxycycline Residue in Soil. Front. Microbiol..

[116] Chen Z., Zhang W., Yang L., Stedtfeld R.D., Peng A., Gu C., Boyd S.A., Li H. (2019). Antibiotic resistance genes and bacterial communities in cornfield and pasture soils receiving swine and dairy manures. Environ. Pollut..

[117] Yang Q., Ren S., Niu T., Guo Y., Qi S., Han X., Liu D., Pan F. (2014). Distribution of antibiotic-resistant bacteria in chicken manure and manure-fertilized vegetables. Environ. Sci. Pollut. Res. Int..

[118] Munir M., Xagoraraki I. (2011). Levels of antibiotic resistance genes in manure, biosolids, and fertilized soil. J. Environ. Qual..

[119] (2013). Eurostat. Archive: Agri-Environmental Indicator—Manure Application.

[120] Zhang Y., Gu A., Cen T., Li X., He M., Li D., Chen J.-M. (2018). Sub-inhibitory concentrations of heavy metals facilitate the horizontal transfer of plasmid-mediated antibiotic resistance genes in water environment. Environ. Pollut..

[121] Kang C.-H., So J.-S. (2016). Heavy metal and antibiotic resistance of ureolytic bacteria and their immobilization of heavy metals. Ecol. Eng..

[122] Gorovtsov A.V., Sazykin I.S., Sazykina M.A. (2018). The influence of heavy metals, polyaromatic hydrocarbons, and polychlorinated biphenyls pollution on the development of antibiotic resistance in soils. Environ. Sci. Pollut. Res. Int..

[123] Zhao Z., Wang J., Han Y., Chen J., Liu G., Lu H., Yan B., Chen S. (2017). Nutrients, heavy metals and microbial communities co-driven distribution of antibiotic resistance genes in adjacent environment of mariculture. Environ. Pollut..

[124] Chen Q.L., Zhu D., An X.L., Ding J., Zhu Y.G., Cui L. (2019). Does nano silver promote the selection of antibiotic resistance genes in soil and plant?. Environ. Int..

[125] Yang Y., Xu C., Cao X., Lin H., Wang J. (2017). Antibiotic resistance genes in surface water of eutrophic urban lakes are related to heavy metals, antibiotics, lake morphology and anthropic impact. Ecotoxicology.

[126] Ji X., Shen Q., Liu F., Ma J., Xu G., Wang Y., Wu M. (2012). Antibiotic resistance gene abundances associated with antibiotics and heavy metals in animal manures and agricultural soils adjacent to feedlots in Shanghai, China. J. Hazard. Mater..

[127] Lu L., Liu J., Li Z., Zou X., Guo J., Liu Z., Yang J., Zhou Y. (2020). Antibiotic resistance gene abundances associated with heavy metals and antibiotics in the sediments of Changshou Lake in the three Gorges Reservoir area, China. Ecol. Indic..

[128] Zhou B., Wang C., Zhao Q., Wang Y., Huo M., Wang J., Wang S. (2016). Prevalence and dissemination of antibiotic resistance genes and coselection of heavy metals in Chinese dairy farms. J. Hazard. Mater..

[129] Li H., Tian Y., Liu W., Long Y., Ye J., Li B., Li N., Yan M., Zhu C. (2020). Impact of electrokinetic remediation of heavy metal contamination on antibiotic resistance in soil. Chem. Eng. J..

[130] Mazhar S.H., Li X., Rashid A., Su J., Xu J., Brejnrod A.D., Su J.Q., Wu Y., Zhu Y.G., Zhou S.G. (2021). Co-selection of antibiotic resistance genes, and mobile genetic elements in the presence of heavy metals in poultry farm environments. Sci. Total Environ..

[131] Nguyen C.C., Hugie C.N., Kile M.L., Navab-Daneshmand T. (2019). Association between heavy metals and antibiotic-resistant human pathogens in environmental reservoirs: A review. Front. Environ. Sci. Eng..

[132] Seiler C., Berendonk T.U. (2012). Heavy metal driven co-selection of antibiotic resistance in soil and water bodies impacted by agriculture and aquaculture. Front. Microbiol..

[133] Safari Sinegani A.A., Younessi N. (2017). Antibiotic resistance of bacteria isolated from heavy metal-polluted soils with different land uses. J. Glob. Antimicrob. Resist..

[134] Yamamura S., Watanabe K., Suda W., Tsuboi S., Watanabe M. (2014). Effect of antibiotics on redox transformations of arsenic and diversity of arsenite-oxidizing bacteria in sediment microbial communities. Environ. Sci. Technol..

[135] Chen S., Li X., Sun G., Zhang Y., Su J., Ye J. (2015). Heavy Metal Induced Antibiotic Resistance in Bacterium LSJC7. Int. J. Mol. Sci..

[136] Li X., Gu A.Z., Zhang Y., Xie B., Li D., Chen J. (2019). Sub-lethal concentrations of heavy metals induce antibiotic resistance via mutagenesis. J. Hazard. Mater..

[137] Zhang F., Zhao X., Li Q., Liu J., Ding J., Wu H., Zhao Z., Ba Y., Cheng X., Cui L. (2018). Bacterial community structure and abundances of antibiotic resistance genes in heavy metals contaminated agricultural soil. Environ. Sci. Pollut. Res. Int..

[138] Xing B.S., Jin R.C. (2018). Inhibitory effects of heavy metals and antibiotics on nitrifying bacterial activities in mature partial nitritation. Chemosphere.

[139] Benghait Y., Blaghen M. (2020). Heavy metals and antibiotics resistance of bacteria isolated from Marchica lagoon: Biodegradation of anthracene on submerged aerated fixed bed reactor. Environ. Technol..

[140] Nourhene S., Rihab L., Fethi B.A., Karima B.R., Amina B. (2013). Slime producing, heavy metals and antibiotics resistance in Aeromonas hydrophila isolated in Tunisia. Afr. J. Microbiol. Res..

[141] Oyetibo G.O., Ilori M.O., Adebusoye S.A., Obayori O.S., Amund O.O. (2010). Bacteria with dual resistance to elevated concentrations of heavy metals and antibiotics in Nigerian contaminated systems. Environ. Monit. Assess..

[142] Yamina B., Tahar B., Marie Laure F. (2012). Isolation and screening of heavy metal resistant bacteria from wastewater: A study of heavy metal co-resistance and antibiotics resistance. Water Sci. Technol..

[143] Zhou Y., Xu Y.B., Xu J.X., Zhang X.H., Xu S.H., Du Q.P. (2015). Combined toxic effects of heavy metals and antibiotics on a Pseudomonas fluorescens strain ZY2 isolated from swine wastewater. Int. J. Mol. Sci..

[144] Abdelrehim K., Soltan E.-S., Abu-Garbia M., El-Zien F. (2014). Heavy Metals and Antibiotics Resistance of Halophilic Bacteria Isolated from Different Areas in Red Sea, Egypt. Egypt. Acad. J. Biol. Sci. G Microbiol..

[145] Bhardwaj R., Gupta A., Garg J.K. (2018). Impact of heavy metals on inhibitory concentration of *Escherichia coli*-a case study of river Yamuna system, Delhi, India. Environ. Monit. Assess..

[146] Sharifi Y., Abedzadeh A., Salighe A., Kalhor N. (2015). Antibiotics and heavy metals resistance patterns of Enterococcus faecalis and faecium bacteria isolated from the human and the livestock sources. Environ. Health Eng. Manag. J..

[147] Adekanmbi A.O., Falodun O.I. (2015). Heavy Metals and Antibiotics Susceptibility Profiles of *Staphylococcus aureus*; Isolated from Several Points Receiving Daily Input from the Bodija Abattoir in Ibadan, Oyo State, Nigeria. Adv. Microbiol..

[148] Li G., Sun G.X., Ren Y., Luo X.S., Zhu Y.G. (2018). Urban soil and human health: A review. Eur. J. Soil Sci..

[149] Zhou C., Pan Y., Ge S., Coulon F., Yang Z. (2021). Rapid methods for antimicrobial resistance diagnosis in contaminated soils for effective remediation strategy. TrAC Trends Anal. Chem..

[150] Wang J., Wang J., Zhao Z., Chen J., Lu H., Liu G., Zhou J., Guan X. (2017). PAHs accelerate the propagation of antibiotic resistance genes in coastal water microbial community. Environ. Pollut..

[151] Chen B., He R., Yuan K., Chen E., Lin L., Chen X., Sha R., Zhong J., Lin L., Yang L. (2016). Polycyclic aromatic hydrocarbons (PAHs) enriching antibiotic resistance genes (ARGs) in the soils. Environ. Pollut..

[152] Amala S.E., Agwor N.O., Vivian Agi N., Monsi T.P. (2021). Evaluation of the Impact of Hydrocarbon-Generated Soot on Antibiotics Susceptibility of *Staphylococcus aureus* and *Escherichia coli* Isolates. Adv. Microbiol..

[153] Cunningham C., Kuyukina M., Ivshina I., Konev A., Peshkur T., Knapp C. (2020). Potential risks of antibiotic resistant bacteria and genes in bioremediation of petroleum hydrocarbon contaminated soils. Environ. Sci. Processes Impacts.

[154] Das N., Kotoky R., Maurya A.P., Bhuyan B., Pandey P. (2021). Paradigm shift in antibiotic-resistome of petroleum hydrocarbon contaminated soil. Sci. Total Environ..

[155] Hemala L., Zhang D., Margesin R. (2014). Cold-active antibacterial and antifungal activities and antibiotic resistance of bacteria isolated from an alpine hydrocarbon-contaminated industrial site. Res. Microbiol..

[156] Wetherill Y.B., Akingbemi B.T., Kanno J., McLachlan J.A., Nadal A., Sonnenschein C., Watson C.S., Zoeller R.T., Belcher S.M. (2007). In vitro molecular mechanisms of bisphenol A action. Reprod. Toxicol..

[157] Vandenberg L.N., Hauser R., Marcus M., Olea N., Welshons W.V. (2007). Human exposure to bisphenol A (BPA). Reprod. Toxicol..

[158] Rochester J.R. (2013). Bisphenol A and human health: A review of the literature. Reprod. Toxicol..

[159] Pop C.-E., Draga S., Măciucă R., Niță R., Crăciun N., Wolff R. (2021). Bisphenol a Effects in Aqueous Environment on *Lemna minor*. Processes.

[160] Eladak S., Grisin T., Moison D., Guerquin M.J., N’Tumba-Byn T., Pozzi-Gaudin S., Benachi A., Livera G., Rouiller-Fabre V., Habert R. (2015). A new chapter in the bisphenol A story: Bisphenol S and bisphenol F are not safe alternatives to this compound. Fertil. Steril..

[161] Russell A.D. (2000). Do Biocides Select for Antibiotic Resistance?. J. Pharm. Pharmacol..

[162] Hartmann E.M., Hickey R., Hsu T., Betancourt Roman C.M., Chen J., Schwager R., Kline J., Brown G.Z., Halden R.U., Huttenhower C. (2016). Antimicrobial Chemicals Are Associated with Elevated Antibiotic Resistance Genes in the Indoor Dust Microbiome. Environ. Sci. Technol..

[163] Reichert G., Hilgert S., Fuchs S., Azevedo J.C.R. (2019). Emerging contaminants and antibiotic resistance in the different environmental matrices of Latin America. Environ. Pollut..

[164] Maurya A.P., Rajkumari J., Pandey P. (2021). Enrichment of antibiotic resistance genes (ARGs) in polyaromatic hydrocarbon-contaminated soils: A major challenge for environmental health. Environ. Sci. Pollut. Res. Int..

[165] European Environment Agency Overview of Contaminants Affecting Soil and Groundwater in Europe. https://www.eea.europa.eu/data-and-maps/figures/overview-of-contaminants-affecting-soil-and-groundwater-in-europe.

[166] Ya H., Jiang B., Xing Y., Zhang T., Lv M., Wang X. (2021). Recent advances on ecological effects of microplastics on soil environment. Sci. Total Environ..

[167] Zhang Y., Lu J., Wu J., Wang J., Luo Y. (2020). Potential risks of microplastics combined with superbugs: Enrichment of antibiotic resistant bacteria on the surface of microplastics in mariculture system. Ecotoxicol. Environ. Saf..

[168] Liu Y., Liu W., Yang X., Wang J., Lin H., Yang Y. (2021). Microplastics are a hotspot for antibiotic resistance genes: Progress and perspective. Sci. Total Environ..

[169] Kumar M., Xiong X., He M., Tsang D.C.W., Gupta J., Khan E., Harrad S., Hou D., Ok Y.S., Bolan N.S. (2020). Microplastics as pollutants in agricultural soils. Environ. Pollut..

[170] Ma J., Sheng G.D., O’Connor P. (2020). Microplastics combined with tetracycline in soils facilitate the formation of antibiotic resistance in the Enchytraeus crypticus microbiome. Environ. Pollut..

[171] Yan X., Yang X., Tang Z., Fu J., Chen F., Zhao Y., Ruan L., Yang Y. (2020). Downward transport of naturally-aged light microplastics in natural loamy sand and the implication to the dissemination of antibiotic resistance genes. Environ. Pollut..

[172] Huang F.Y., Yang K., Zhang Z.X., Su J.Q., Zhu Y.G., Zhang X. (2019). Effects of Microplastics on Antibiotic Resistance Genes in Estuarine Sediments. Huan Jing Ke Xue.

[173] Peng C., Zhang X., Zhang X., Liu C., Chen Z., Sun H., Wang L. (2021). Bacterial Community under the Influence of Microplastics in Indoor Environment and the Health Hazards Associated with Antibiotic Resistance Genes. Environ. Sci. Technol..

[174] Wang Y., Wang X., Li Y., Liu Y., Sun Y., Xia S., Zhao J. (2021). Effects of coexistence of tetracycline, copper and microplastics on the fate of antibiotic resistance genes in manured soil. Sci. Total Environ..

[175] Shi J., Wu D., Su Y., Xie B. (2021). Selective enrichment of antibiotic resistance genes and pathogens on polystyrene microplastics in landfill leachate. Sci. Total Environ..

[176] Sucato A., Vecchioni L., Savoca D., Presentato A., Arculeo M., Alduina R. (2021). A Comparative Analysis of Aquatic and Polyethylene-Associated Antibiotic-Resistant Microbiota in the Mediterranean Sea. Biology.

[177] Zhu D., Ma J., Li G., Rillig M.C., Zhu Y.G. (2021). Soil plastispheres as hotpots of antibiotic resistance genes and potential pathogens. ISME J..

[178] Sathicq M.B., Sabatino R., Corno G., Di Cesare A. (2021). Are microplastic particles a hotspot for the spread and the persistence of antibiotic resistance in aquatic systems?. Environ. Pollut..

[179] Imran M., Das K.R., Naik M.M. (2019). Co-selection of multi-antibiotic resistance in bacterial pathogens in metal and microplastic contaminated environments: An emerging health threat. Chemosphere.

[180] Gao D., Li X.Y., Liu H.T. (2020). Source, occurrence, migration and potential environmental risk of microplastics in sewage sludge and during sludge amendment to soil. Sci. Total Environ..

[181] Zhu F., Zhu C., Wang C., Gu C. (2019). Occurrence and Ecological Impacts of Microplastics in Soil Systems: A Review. Bull. Environ. Contam. Toxicol..

[182] Zhang B., Yang X., Chen L., Chao J., Teng J., Wang Q. (2020). Microplastics in soils: A review of possible sources, analytical methods and ecological impacts. J. Chem. Technol. Biotechnol..

[183] Cerqueira F., Christou A., Fatta-Kassinos D., Vila-Costa M., Bayona J.M., Pina B. (2020). Effects of prescription antibiotics on soil- and root-associated microbiomes and resistomes in an agricultural context. J. Hazard. Mater..

[184] Urra J., Alkorta I., Mijangos I., Epelde L., Garbisu C. (2019). Application of sewage sludge to agricultural soil increases the abundance of antibiotic resistance genes without altering the composition of prokaryotic communities. Sci. Total Environ..

[185] Xu S., Lu W., Qasim M.Z. (2020). High-throughput characterization of the expressed antibiotic resistance genes in sewage sludge with transcriptional analysis. Ecotoxicol. Environ. Saf..

[186] Venkatesan A.K., Done H.Y., Halden R.U. (2015). United States National Sewage Sludge Repository at Arizona State University—A new resource and research tool for environmental scientists, engineers, and epidemiologists. Environ. Sci. Pollut. Res. Int..

[187] Poszytek K., Karczewska-Golec J., Ciok A., Decewicz P., Dziurzynski M., Gorecki A., Jakusz G., Krucon T., Lomza P., Romaniuk K. (2018). Genome-Guided Characterization of Ochrobactrum sp. POC9 Enhancing Sewage Sludge Utilization-Biotechnological Potential and Biosafety Considerations. Int. J. Environ. Res. Public Health.

[188] Urra J., Alkorta I., Mijangos I., Garbisu C. (2018). Data on links between structural and functional prokaryotic diversity in long-term sewage sludge amended soil. Data Brief..

[189] Karkman A., Do T.T., Walsh F., Virta M.P.J. (2018). Antibiotic-Resistance Genes in Waste Water. Trends Microbiol..

[190] Yang Y., Li B., Zou S., Fang H.H.P., Zhang T. (2014). Fate of antibiotic resistance genes in sewage treatment plant revealed by metagenomic approach. Water Res..

[191] Rahube T.O., Marti R., Scott A., Tien Y.C., Murray R., Sabourin L., Duenk P., Lapen D.R., Topp E. (2016). Persistence of antibiotic resistance and plasmid-associated genes in soil following application of sewage sludge and abundance on vegetables at harvest. Can. J. Microbiol..

[192] Lyu J., Yang L., Zhang L., Ye B., Wang L. (2020). Antibiotics in soil and water in China-a systematic review and source analysis. Environ. Pollut..

[193] Gatica J., Cytryn E. (2013). Impact of treated wastewater irrigation on antibiotic resistance in the soil microbiome. Environ. Sci. Pollut. Res. Int..

[194] Kummerer K. (2003). Significance of antibiotics in the environment. J. Antimicrob. Chemother..

[195] Khan N.A., Ahmed S., Farooqi I.H., Ali I., Vambol V., Changani F., Yousefi M., Vambol S., Khan S.U., Khan A.H. (2020). Occurrence, sources and conventional treatment techniques for various antibiotics present in hospital wastewaters: A critical review. TrAC Trends Anal. Chem..

[196] Hubeny J., Harnisz M., Korzeniewska E., Buta M., Zielinski W., Rolbiecki D., Giebultowicz J., Nalecz-Jawecki G., Plaza G. (2021). Industrialization as a source of heavy metals and antibiotics which can enhance the antibiotic resistance in wastewater, sewage sludge and river water. PLoS ONE.

[197] Rahube T.O., Marti R., Scott A., Tien Y.C., Murray R., Sabourin L., Zhang Y., Duenk P., Lapen D.R., Topp E. (2014). Impact of fertilizing with raw or anaerobically digested sewage sludge on the abundance of antibiotic-resistant coliforms, antibiotic resistance genes, and pathogenic bacteria in soil and on vegetables at harvest. Appl. Environ. Microbiol..

[198] Zarfel G., Galler H., Feierl G., Haas D., Kittinger C., Leitner E., Grisold A.J., Mascher F., Posch J., Pertschy B. (2013). Comparison of extended-spectrum-beta-lactamase (ESBL) carrying *Escherichia coli* from sewage sludge and human urinary tract infection. Environ. Pollut..

[199] Sahlstrom L., Rehbinder V., Albihn A., Aspan A., Bengtsson B. (2009). Vancomycin resistant enterococci (VRE) in Swedish sewage sludge. Acta Vet. Scand..

[200] Markowicz A., Bondarczuk K., Wiekiera A., Sułowicz S. (2021). Is sewage sludge a valuable fertilizer? A soil microbiome and resistome study under field conditions. J. Soils Sediments.

[201] Buta M., Hubeny J., Zielinski W., Harnisz M., Korzeniewska E. (2021). Sewage sludge in agriculture—The effects of selected chemical pollutants and emerging genetic resistance determinants on the quality of soil and crops—A review. Ecotoxicol. Environ. Saf..

[202] You R., Margenat A., Lanzas C.S., Canameras N., Carazo N., Navarro-Martin L., Matamoros V., Bayona J.M., Diez S. (2020). Dose effect of Zn and Cu in sludge-amended soils on vegetable uptake of trace elements, antibiotics, and antibiotic resistance genes: Human health implications. Environ. Res..

[203] Cucina M., Ricci A., Zadra C., Pezzolla D., Tacconi C., Sordi S., Gigliotti G. (2019). Benefits and risks of long-term recycling of pharmaceutical sewage sludge on agricultural soil. Sci. Total Environ..

[204] Bondarczuk K., Markowicz A., Piotrowska-Seget Z. (2016). The urgent need for risk assessment on the antibiotic resistance spread via sewage sludge land application. Environ. Int..

[205] Collivignarelli M., Abbà A., Frattarola A., Carnevale Miino M., Padovani S., Katsoyiannis I., Torretta V. (2019). Legislation for the Reuse of Biosolids on Agricultural Land in Europe: Overview. Sustainability.

[206] Fijalkowski K., Rorat A., Grobelak A., Kacprzak M.J. (2017). The presence of contaminations in sewage sludge—The current situation. J. Environ. Manage..

[207] Rad A.K., Shamshiri R.R., Azarm H., Balasundram S.K., Sultan M. (2021). Effects of the COVID-19 Pandemic on Food Security and Agriculture in Iran: A Survey. Sustainability.

[208] Yan W., Bai R., Wang S., Tian X., Li Y., Wang S., Yang F., Xiao Y., Lu X., Zhao F. (2020). Antibiotic resistance genes are increased by combined exposure to sulfamethoxazole and naproxen but relieved by low-salinity. Environ. Int..

[209] Tan L., Wang F., Liang M., Wang X., Das R., Mao D., Luo Y. (2019). Antibiotic resistance genes attenuated with salt accumulation in saline soil. J. Hazard. Mater..

[210] Sepulveda-Correa A., Daza-Giraldo L.V., Polania J., Arenas N.E., Munoz-Garcia A., Sandoval-Figueredo A.V., Vanegas J. (2021). Genes associated with antibiotic tolerance and synthesis of antimicrobial compounds in a mangrove with contrasting salinities. Mar. Pollut. Bull..

[211] Liu M., Li Q., Sun H., Jia S., He X., Li M., Zhang X.-X., Ye L. (2018). Impact of salinity on antibiotic resistance genes in wastewater treatment bioreactors. Chem. Eng. J..

[212] Chen Y., Zhou J.L., Cheng L., Zheng Y.Y., Xu J. (2017). Sediment and salinity effects on the bioaccumulation of sulfamethoxazole in zebrafish (Danio rerio). Chemosphere.

[213] Yang C.-C., Huang C.-L., Cheng T.-C., Lai H.-T. (2015). Inhibitory effect of salinity on the photocatalytic degradation of three sulfonamide antibiotics. Int. Biodeterior. Biodegrad..

[214] Nayiga S., Kayendeke M., Nabirye C., Willis L.D., Chandler C.I.R., Staedke S.G. (2020). Use of antibiotics to treat humans and animals in Uganda: A cross-sectional survey of households and farmers in rural, urban and peri-urban settings. JAC Antimicrob. Resist..

[215] Madaras-Kelly K. (2003). Optimizing antibiotic use in hospitals: The role of population-based antibiotic surveillance in limiting antibiotic resistance. Insights from the society of infectious diseases pharmacists. Pharmacotherapy.

[216] Kromker V., Leimbach S. (2017). Mastitis Treatment-Reduction in antibiotic usage in dairy cows. Reprod. Domest. Anim..

[217] Levy S. (2014). Reduced antibiotic use in livestock: How Denmark tackled resistance. Environ. Health Perspect..

[218] Mevius D., Heederik D. (2014). Reduction of antibiotic use in animals “let’s go Dutch”. J. Verbrauch. Lebensm..

[219] Sneeringer S., Short G., MacLachlan M., Bowman M. (2020). Impacts on Livestock Producers and Veterinarians of FDA Policies on Use of Medically Important Antibiotics in Food Animal Production. Appl. Econ. Perspect. Policy.

[220] Founou L.L., Founou R.C., Essack S.Y. (2016). Antibiotic Resistance in the Food Chain: A Developing Country-Perspective. Front. Microbiol..

[221] Lhermie G., Grohn Y.T., Raboisson D. (2016). Addressing Antimicrobial Resistance: An Overview of Priority Actions to Prevent Suboptimal Antimicrobial Use in Food-Animal Production. Front. Microbiol..

[222] Samanta I., Bandyopadhyay S. (2020). The emergence of antimicrobial-resistant bacteria in livestock, poultry and agriculture. Antimicrobial Resistance in Agriculture.

[223] Mshana S.E., Sindato C., Matee M.I., Mboera L.E.G. (2021). Antimicrobial Use and Resistance in Agriculture and Food Production Systems in Africa: A Systematic Review. Antibiotics.

[224] Robert P.C. (2002). Precision agriculture: A challenge for crop nutrition management. Plant Soil.

[225] Bongiovanni R., Lowenberg-Deboer J. (2004). Precision Agriculture and Sustainability. Precis. Agric..

[226] Zarei M., Kaviani Rad A. (2020). Covid-19, Challenges and Recommendations in Agriculture. J. Bot. Res..

[227] Du L., Liu W. (2011). Occurrence, fate, and ecotoxicity of antibiotics in agro-ecosystems. A review. Agron. Sustain. Dev..

[228] Sodhi K.K., Kumar M., Singh D.K. (2021). Insight into the amoxicillin resistance, ecotoxicity, and remediation strategies. J. Water Process. Eng..

[229] Gu S., Kang X., Wang L., Lichtfouse E., Wang C. (2018). Clay mineral adsorbents for heavy metal removal from wastewater: A review. Environ. Chem. Lett..

[230] Han H., Rafiq M.K., Zhou T., Xu R., Masek O., Li X. (2019). A critical review of clay-based composites with enhanced adsorption performance for metal and organic pollutants. J. Hazard. Mater..

[231] Adeyemo A.A., Adeoye I.O., Bello O.S. (2015). Adsorption of dyes using different types of clay: A review. Appl. Water Sci..

[232] Mustapha S., Ndamitso M.M., Abdulkareem A.S., Tijani J.O., Mohammed A.K., Shuaib D.T. (2019). Potential of using kaolin as a natural adsorbent for the removal of pollutants from tannery wastewater. Heliyon.

[233] Yang W., Wu Y., Zhang L., Jiang J., Feng L. (2014). Removal of five selected pharmaceuticals by coagulation in the presence of dissolved humic acids and kaolin. Desalination Water Treat..

[234] Ugwu I.M., Igbokwe O.A. (2019). Sorption of heavy metals on clay minerals and oxides: A review. Adv. Sorpt. Process Appl..

[235] Al-Mashhadany D.A. (2019). Detection of antibiotic residues among raw beef in Erbil City (Iraq) and impact of temperature on antibiotic remains. Ital. J. Food Saf..

[236] Zhang Y., Liu H., Dai X., Cai C., Wang J., Wang M., Shen Y., Wang P. (2020). Impact of application of heat-activated persulfate oxidation treated erythromycin fermentation residue as a soil amendment: Soil chemical properties and antibiotic resistance. Sci. Total Environ..

[237] Xu R., Zhang Y., Xiong W., Sun W., Fan Q., Zhaohui Y. (2020). Metagenomic approach reveals the fate of antibiotic resistance genes in a temperature-raising anaerobic digester treating municipal sewage sludge. J. Clean. Prod..

[238] Sun C., Li W., Chen Z., Qin W., Wen X. (2019). Responses of antibiotics, antibiotic resistance genes, and mobile genetic elements in sewage sludge to thermal hydrolysis pre-treatment and various anaerobic digestion conditions. Environ. Int..

[239] Liao H., Lu X., Rensing C., Friman V.P., Geisen S., Chen Z., Yu Z., Wei Z., Zhou S., Zhu Y. (2018). Hyperthermophilic Composting Accelerates the Removal of Antibiotic Resistance Genes and Mobile Genetic Elements in Sewage Sludge. Environ. Sci. Technol..

[240] Deng W., Zhang A., Chen S., He X., Jin L., Yu X., Yang S., Li B., Fan L., Ji L. (2020). Heavy metals, antibiotics and nutrients affect the bacterial community and resistance genes in chicken manure composting and fertilized soil. J. Environ. Manag..

[241] Gou M., Hu H.W., Zhang Y.J., Wang J.T., Hayden H., Tang Y.Q., He J.Z. (2018). Aerobic composting reduces antibiotic resistance genes in cattle manure and the resistome dissemination in agricultural soils. Sci. Total Environ..

[242] Youngquist C.P., Mitchell S.M., Cogger C.G. (2016). Fate of Antibiotics and Antibiotic Resistance during Digestion and Composting: A Review. J. Environ. Qual..

[243] Keenum I., Williams R.K., Ray P., Garner E.D., Knowlton K.F., Pruden A. (2021). Combined effects of composting and antibiotic administration on cattle manure-borne antibiotic resistance genes. Microbiome.

[244] Sardar M.F., Zhu C., Geng B., Ahmad H.R., Song T., Li H. (2021). The fate of antibiotic resistance genes in cow manure composting: Shaped by temperature-controlled composting stages. Bioresour. Technol..

[245] Gao M., Qiu T., Sun Y., Wang X. (2018). The abundance and diversity of antibiotic resistance genes in the atmospheric environment of composting plants. Environ. Int..

[246] Guerin T.F. (2000). The differential removal of aged polycyclic aromatic hydrocarbons from soil during bioremediation. Environ. Sci. Pollut. Res..

[247] Imam A., Suman S.K., Ghosh D., Kanaujia P.K. (2019). Analytical approaches used in monitoring the bioremediation of hydrocarbons in petroleum-contaminated soil and sludge. TrAC Trends Anal. Chem..

[248] Stroud J.L., Paton G.I., Semple K.T. (2007). Microbe-aliphatic hydrocarbon interactions in soil: Implications for biodegradation and bioremediation. J. Appl. Microbiol..

[249] Diplock E.E., Mardlin D.P., Killham K.S., Paton G.I. (2009). Predicting bioremediation of hydrocarbons: Laboratory to field scale. Environ. Pollut..

[250] Ghazali F.M., Rahman R.N.Z.A., Salleh A.B., Basri M. (2004). Biodegradation of hydrocarbons in soil by microbial consortium. Int. Biodeterior. Biodegrad..

[251] Gargouri B., Karray F., Mhiri N., Aloui F., Sayadi S. (2014). Bioremediation of petroleum hydrocarbons-contaminated soil by bacterial consortium isolated from an industrial wastewater treatment plant. J. Chem. Technol. Biotechnol..

[252] Guarino C., Spada V., Sciarrillo R. (2017). Assessment of three approaches of bioremediation (Natural Attenuation, Landfarming and Bioagumentation—Assistited Landfarming) for a petroleum hydrocarbons contaminated soil. Chemosphere.

[253] Muyzer G., Stams A.J. (2008). The ecology and biotechnology of sulphate-reducing bacteria. Nat. Rev. Microbiol..

[254] Castro H.F., Williams N.H., Ogram A. (2000). Phylogeny of sulfate-reducing bacteria1. FEMS Microbiol. Ecol..

[255] Xu Y.N., Chen Y. (2020). Advances in heavy metal removal by sulfate-reducing bacteria. Water Sci. Technol..

[256] Jong T., Parry D.L. (2003). Removal of sulfate and heavy metals by sulfate reducing bacteria in short-term bench scale upflow anaerobic packed bed reactor runs. Water Res..

[257] Zhao Q., Li X., Xiao S., Peng W., Fan W. (2021). Integrated remediation of sulfate reducing bacteria and nano zero valent iron on cadmium contaminated sediments. J. Hazard. Mater..

[258] Barton L.L., Fauque G.D. (2009). Biochemistry, Physiology and Biotechnology of Sulfate-Reducing Bacteria. Adv. Appl. Microbiol..

[259] Fang H., Oberoi A.S., He Z., Khanal S.K., Lu H. (2021). Ciprofloxacin-degrading Paraclostridium sp. isolated from sulfate-reducing bacteria-enriched sludge: Optimization and mechanism. Water Res..

[260] Zhang H., Song S., Jia Y., Wu D., Lu H. (2019). Stress-responses of activated sludge and anaerobic sulfate-reducing bacteria sludge under long-term ciprofloxacin exposure. Water Res..

[261] Zhang H., Jia Y., Khanal S.K., Lu H., Fang H., Zhao Q. (2018). Understanding the Role of Extracellular Polymeric Substances on Ciprofloxacin Adsorption in Aerobic Sludge, Anaerobic Sludge, and Sulfate-Reducing Bacteria Sludge Systems. Environ. Sci. Technol..

[262] Jia Y., Khanal S.K., Shu H., Zhang H., Chen G.H., Lu H. (2018). Ciprofloxacin degradation in anaerobic sulfate-reducing bacteria (SRB) sludge system: Mechanism and pathways. Water Res..

[263] Zhang X., Zhu R., Li W., Ma J., Lin H. (2021). Genomic insights into the antibiotic resistance pattern of the tetracycline-degrading bacterium, Arthrobacter nicotianae OTC-16. Sci. Rep..

[264] Maki T., Hasegawa H., Kitami H., Fumoto K., Munekage Y., Ueda K. (2006). Bacterial degradation of antibiotic residues in marine fish farm sediments of Uranouchi Bay and phylogenetic analysis of antibiotic-degrading bacteria using 16S rDNA sequences. Fish. Sci..

[265] Hirth N., Topp E., Dörfler U., Stupperich E., Munch J.C., Schroll R. (2016). An effective bioremediation approach for enhanced microbial degradation of the veterinary antibiotic sulfamethazine in an agricultural soil. Chem. Biol. Technol. Agric..

[266] Mojiri A., Baharlooeian M., Zahed M.A. (2021). The Potential of Chaetoceros muelleri in Bioremediation of Antibiotics: Performance and Optimization. Int. J. Environ. Res. Public Health.

[267] Wu Y., Feng P., Li R., Chen X., Li X., Sumpradit T., Liu P. (2019). Progress in microbial remediation of antibiotic-residue contaminated environment. Sheng Wu Gong Cheng Xue Bao.

[268] Xue W., Zhou Q., Li F. (2019). Bacterial community changes and antibiotic resistance gene quantification in microbial electrolysis cells during long-term sulfamethoxazole treatment. Bioresour. Technol..

[269] Hua T., Li S., Li F., Ondon B.S., Liu Y., Wang H. (2019). Degradation performance and microbial community analysis of microbial electrolysis cells for erythromycin wastewater treatment. Biochem. Eng. J..

[270] Zhang X., Li R. (2020). Variation and distribution of antibiotic resistance genes and their potential hosts in microbial electrolysis cells treating sewage sludge. Bioresour. Technol..

[271] Zhang X., Li R. (2020). Electrodes bioaugmentation promotes the removal of antibiotics from concentrated sludge in microbial electrolysis cells. Sci. Total Environ..

[272] Yan W., Xiao Y., Yan W., Ding R., Wang S., Zhao F. (2019). The effect of bioelectrochemical systems on antibiotics removal and antibiotic resistance genes: A review. Chem. Eng. J..

[273] Dong X., Rao D., Tian L., Wang Q., Yang K. (2020). A slurry microcosm study on the interaction between antibiotics and soil bacterial community. Heliyon.

[274] Naqvi S.A.R., Nadeem S., Komal S., Naqvi S.A.A., Mubarik M.S., Qureshi S.Y., Ahmad S., Abbas A., Zahid M., Raza S.S. (2019). Antioxidants: Natural Antibiotics. Antioxidants.

[275] Abd El-Ghany W.A. (2020). Paraprobiotics and postbiotics: Contemporary and promising natural antibiotics alternatives and their applications in the poultry field. Open Vet. J..

[276] Gupta R., Sharma S. (2020). Herbal antibiotics: A Review. Bull. Env. Pharmacol. Life Sci..

[277] Ionescu M.I. (2018). Are Herbal Products an Alternative to Antibiotics. Bacterial Pathogenesis and Antibacterial Control.

[278] Fit N., Gheorghe R., Rapuntean S., Flore C., Nadas G. (2009). Antibacterial Effect of Essential Vegetal Extracts on *Staphylococcus aureus* Compared to Antibiotics. Not. Bot. Horti Agrobot. Cluj Napoca.

[279] Awan U., Andleeb D.S., Kiyani A., Zafar A., Shafique I., Riaz N., Azhar M.T., Uddin H. (2013). Antibacterial screening of traditional herbal plants and standard antibiotics against some human bacterial pathogens. Pak. J. Pharm. Sci..

[280] Saquib S.A., AlQahtani N.A., Ahmad I., Kader M.A., Al Shahrani S.S., Asiri E.A. (2019). Evaluation and Comparison of Antibacterial Efficacy of Herbal Extracts in Combination with Antibiotics on Periodontal pathobionts: An in vitro Microbiological Study. Antibiotics.

[281] Nweze E.I., Eze E.E. (2009). Justification for the use of Ocimum gratissimum L in herbal medicine and its interaction with disc antibiotics. BMC Complement. Altern. Med..

[282] Sharifi-Rad J. (2016). Herbal Antibiotics: Moving back into the mainstream as an alternative for “Superbugs”. Cell Mol. Biol..

[283] Martin K.W., Ernst E. (2003). Herbal medicines for treatment of bacterial infections: A review of controlled clinical trials. Antimicrob. Chemother..

[284] Zasloff M. (2002). Antimicrobial peptides of multicellular organisms. Nature.

[285] Koczulla R., Bals R. (2003). Antimicrobial Peptides: Current Status and Therapeutic Potential. Drugs.

[286] Reddy K.V., Yedery R.D., Aranha C. (2004). Antimicrobial peptides: Premises and promises. Int. J. Antimicrob. Agents.

[287] Sang Y., Blecha F. (2008). Antimicrobial peptides and bacteriocins: Alternatives to traditional antibiotics. Anim. Health Res. Rev..

[288] Leontiadou H., Mark A.E., Marrink S.J. (2006). Antimicrobial Peptides in Action. J. Am. Chem. Soc..

[289] Fjell C.D., Hiss J.A., Hancock R.E., Schneider G. (2011). Designing antimicrobial peptides: Form follows function. Nat. Rev. Drug Discov..

[290] Malmsten M. (2014). Antimicrobial peptides. Ups. J. Med. Sci..

[291] Wang G., Mishra B., Lau K., Lushnikova T., Golla R., Wang X. (2015). Antimicrobial peptides in 2014. Pharmaceuticals.

[292] Bahar A.A., Ren D. (2013). Antimicrobial peptides. Pharmaceuticals.

[293] Kosciuczuk E.M., Lisowski P., Jarczak J., Strzalkowska N., Jozwik A., Horbanczuk J., Krzyzewski J., Zwierzchowski L., Bagnicka E. (2012). Cathelicidins: Family of antimicrobial peptides. A review. Mol. Biol. Rep..

[294] Tamhankar A.J., Stalsby Lundborg C. (2019). Antimicrobials and Antimicrobial Resistance in the Environment and Its Remediation: A Global One Health Perspective. Int. J. Environ. Res. Public Health.

[295] Rad A., Zarei M., Pourghasemi H., Tiefenbacher J. (2022). The COVID-19 crisis and its consequences for global warming and climate change. Computers in Earth and Environmental Sciences.

[296] Manyi-Loh C., Mamphweli S., Meyer E., Okoh A. (2018). Antibiotic Use in Agriculture and Its Consequential Resistance in Environmental Sources: Potential Public Health Implications. Molecules.

